# DNA Strand Displacement Driven by Host–Guest Interactions

**DOI:** 10.1021/jacs.2c05726

**Published:** 2022-09-05

**Authors:** Dilanka V. D. Walpita Kankanamalage, Jennifer H. T. Tran, Noah Beltrami, Kun Meng, Xiao Zhou, Pravin Pathak, Lyle Isaacs, Alexander L. Burin, Mehnaaz F. Ali, Janarthanan Jayawickramarajah

**Affiliations:** † Department of Chemistry, Tulane University, 2015 Percival Stern Hall, New Orleans, Louisiana 70118, United States; ‡ Department of Chemistry, Xavier University of Louisiana, 1 Drexel Drive, New Orleans, Louisiana 70125, United States; § Department of Chemistry and Biochemistry, University of Maryland, College Park, Maryland 20742, United States

## Abstract

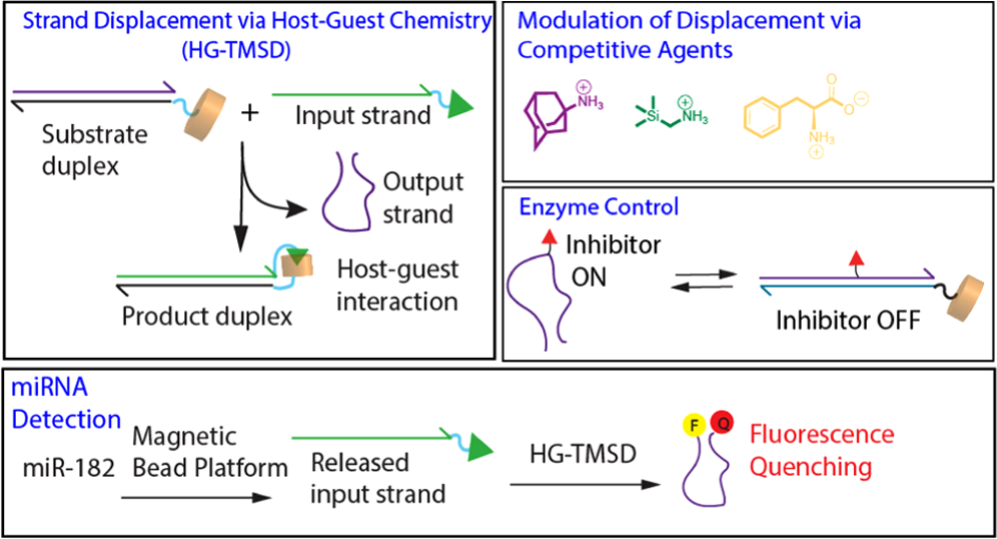

Base-pair-driven toehold-mediated strand displacement (BP-TMSD) is a fundamental concept employed for constructing DNA machines and networks with a gamut of applications—from theranostics to computational devices. To broaden the toolbox of dynamic DNA chemistry, herein, we introduce a synthetic surrogate termed host–guest-driven toehold-mediated strand displacement (HG-TMSD) that utilizes bioorthogonal, cucurbit[7]uril (CB[7]) interactions with guest-linked input sequences. Since control of the strand-displacement process is salient, we demonstrate how HG-TMSD can be finely modulated via changes to the structure of the input sequence (including synthetic guest head-group and/or linker length). Further, for a given input sequence, competing small-molecule guests can serve as effective regulators (with fine and coarse control) of HG-TMSD. To show integration into functional devices, we have incorporated HG-TMSD into machines that control enzyme activity and layered reactions that detect specific microRNA.

## Introduction

**Figure 1:**
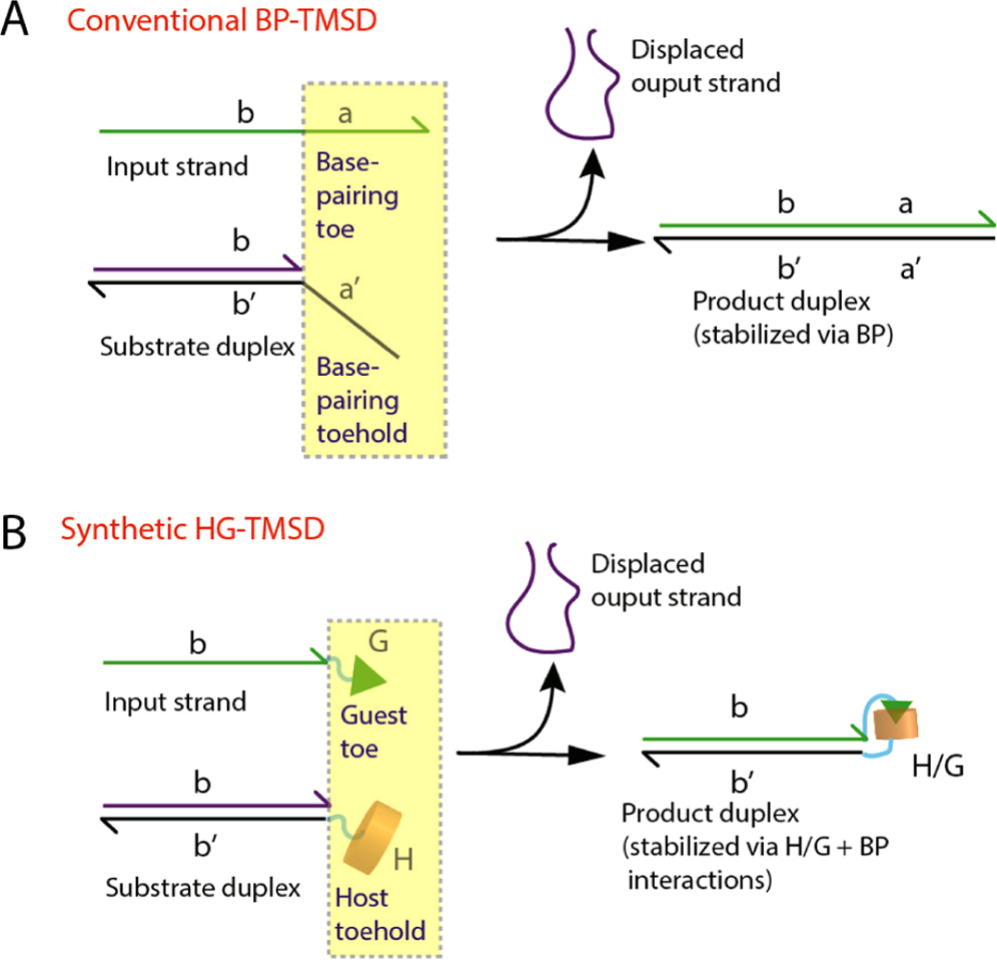
Canonical BP-TMSD versus the novel HG-TMSD introduced in this work. (A) Base-pairing derived toehold/toe pair used for conventional strand displacement (BP-TMSD). The letter labels (i.e., a′, a, b′, b) are stretches of contiguous base-pairing domains that serve as discrete binding units. For example, the dangling toehold domain “a′” on the substrate duplex is complementary to the toe domain “a” of the input strand. These two domains will bind each other leading to the formation of more stable product duplex (that has both a′/a and b′/b involved in base-pairing) and displacement of the shorter output strand. (B) Host–guest (H/G) interaction-driven toehold-mediated strand displacement (HG-TMSD) introduced here. The dangling nucleic acid domains (highlighted in the rectangle in panel A) are replaced with CB[7] host and a synthetic guest such that host–guest (H/G) interactions, followed by DNA base-pairing (b′/b) lead to output strand displacement. The displaced strand can be a labeled reporter (e.g., for fluorescence) or can be used for downstream reactions.

The design of dynamic molecular- and nanomachines and their higher-order interaction networks is a cross-disciplinary research area that has seen tremendous recent growth.^1−4^ In terms of achieving practical applications, such machines need to be coupled to the outside world, and in particular, need to function effectively in aqueous/biological media. In this regard, dynamic DNA chemistry/nanotechnology has led the way generating controlled dynamic systems with potential therapeutic, diagnostic, and computational applications.^5−10^ In particular, the invention of base-pair-driven toehold-mediated strand displacement (BP-TMSD)^11,12^ has served as a founding principle to generate functional DNA-based machines^13−17^—including tweezers,^18,19^ autonomous walkers,^20,21^ molecular diagnostic agents,^17,22−25^ and higher-order networks—that show neural mimicry,^26,27^ control intra/intercell interactions,^28−30^ and perform computational tasks.^31−35^ In BP-TMSD, an invading fuel sequence uses Watson–Crick–Franklin-based toehold/toe interactions to achieve isothermal displacement of an output sequence from a stable duplex substrate (Figure 1A). This system couples an input to the release of a specific output and can be integrated into functional machines and layered reactions. To enhance its scope and applicability, BP-TMSD has been expanded *inter alia* by designing exchange,^36^ remote,^37^ associative,^38^ and allosteric,^39^ toehold systems as well as introducing nucleobase toeholds that are activated by light,^40−42^ enzymes,^43,44^ and metal ions.^45^

Although versatile, the traditional dangling toeholds used in BP-TMSD have notable limitations, including the potential for forming unwanted structures and off-target hybridization and being susceptible to chemical and enzymatic degradation.^46,47^ Further, reducing the length of the DNA domain is salient since longer sequences are costly and have scale-up limitations.^48^ Given these drawbacks of BP-TMSD and, more importantly, to complement BP-TMSD and to introduce novel tools to enhance diversity of the toolbox used to control the function of dynamic DNA machines, it would be a boon to develop synthetic toehold/toe surrogates that are (a) orthogonal to DNA base-pairing, (b) can be readily integrated into oligonucleotides, and (c) the strand displacement can be finely or coarsely controlled by straightforward modulation of the input structure or solution conditions.

With this paper, we introduce the first synthetic toehold/toe system capable of initiating DNA strand displacement by replacement of the base-pairing toehold domain with a supramolecular host (cucurbit[7]uril, CB[7]). This novel host–guest (HG)-driven toehold-mediated strand displacement (HG-TMSD) strategy is illustrated in Figure 1B. We first detail the design of a fluorescence-quenching-based reporter assay that probes the effectiveness of HG-TMSD. Next, we demonstrate how the rate of HG-TMSD can be fine-tuned by introducing structural changes to the input sequence (including modulating linker length and host–guest affinity). We further highlight how, for a given input, HG-TMSD can be additionally controlled (or even turned OFF) by introducing competing small-molecule guests. In addition to establishing the effectiveness and versatility of HG-TMSD, we have also integrated HG-TMSD into (a) functional DNA machines that control protein activity and (b) cascade reactions that enable detection of specific miRNA. Taken together, this work presents a highly versatile supramolecular chemistry approach to strand displacement that is expected to broadly expand the toolbox of dynamic DNA chemistry/nanotechnology.

## Results and Discussion

### Fluorescence-Quenching Reporter Assay to Probe HG-TMSD

**Figure 2:**
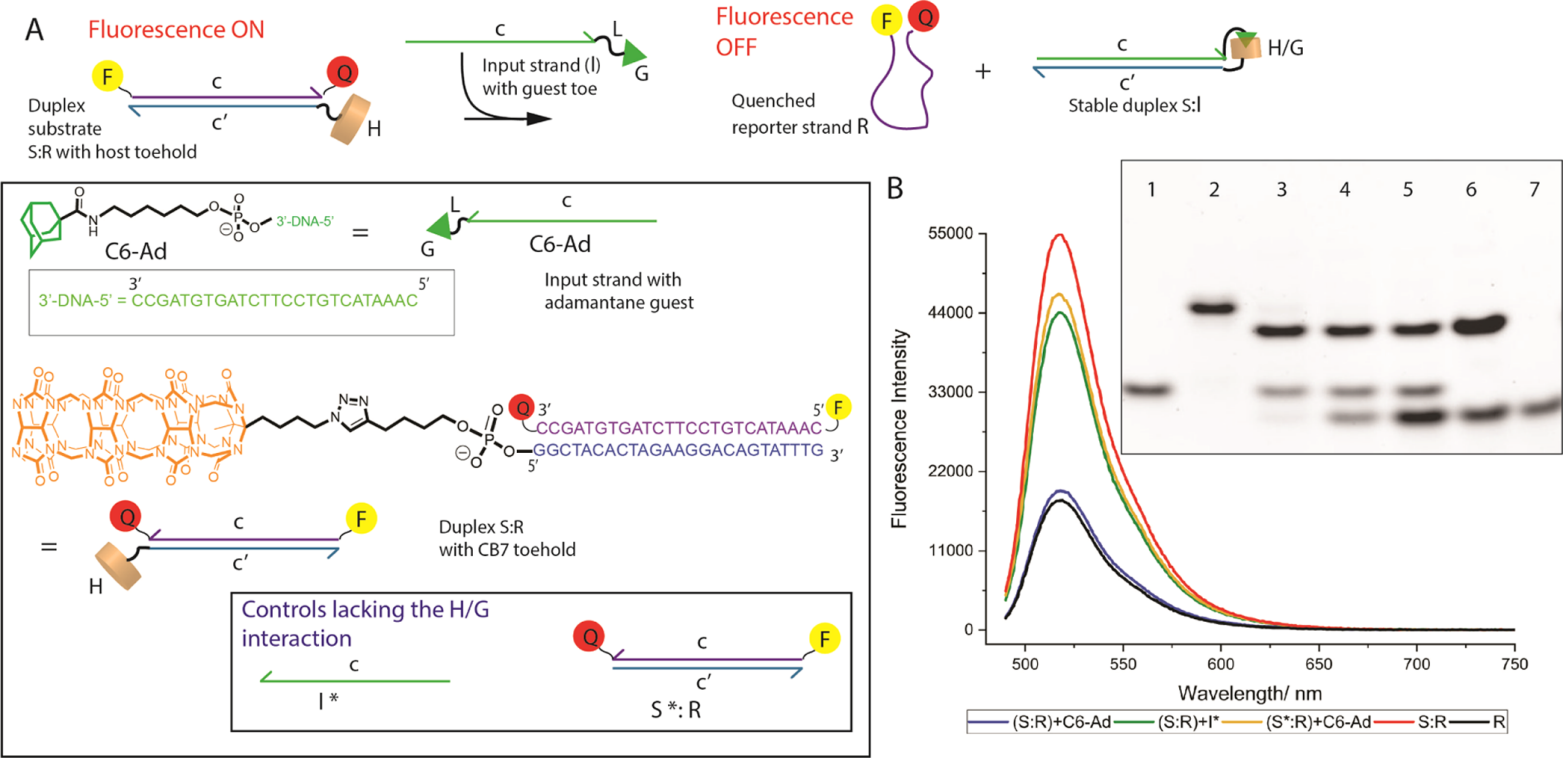
Probing HG-TMSD via fluorescence quenching and polyacrylamide gel electrophoresis (PAGE). (A) Fluorescence reporter assay to probe HG-TMSD. The substrate duplex **S:R** is composed of a reporter strand **R** (containing a 5′-flourescein/3′-dabcyl pair) with sequence c hybridized to substrate **S** with complementary sequence c′. **S** is also functionalized with a CB[7]-host toehold at its 5′ end. The **S:R** duplex is fluorescent because the fluor/quencher pair is separated. Addition of input sequence **I** attached to a guest moiety at its 3′ end (and has core sequence c) should result in HG-TMSD, releasing **R**. Inset: DNA sequences and structures used. H = CB[7], G = adamantane guest, L= hexyl linker, S* = substrate strand without CB[7] host, and I* = input strand without guest unit. (B) Fluorescence emission (in arbitrary units) from the fluorescein unit; **R** alone (black), **S:R** + **C6-Ad** (blue), **S:R** + I* (green), S*:R + **C6-Ad** (yellow), and **S:R** alone (red). The substrate duplex to input strand ratio was 1:10 and the systems were incubated for 18 h at RT prior to data collection. The results are shown as mean of three independent experiments. Buffer: 20 mM Tris, 10 mM NaCl, 5 mM MgCl_2_, pH = 7.5. Inset: Native PAGE showing the effect of input equivalents on HG-TMSD. Here, 1 μM **S:R** was incubated with 1, 5, and 10 μM **C6-Ad** for 18 h at RT. Lanes; 1= **R** only, 2 = **S:R** substrate duplex, 3 = **S:R** + 1 equiv **C6-Ad**, 4 = **S:R** + 5 equiv **C6-Ad**, 5 = **S:R** + 10 equiv **C6-Ad**, 6 = premade **S:I** duplex control using **S** + excess **C6-Ad**, and 7 = **C6-Ad** only. PAGE was carried out in TBE buffer at 95 V for 5 h at RT.

The CB[7]–guest interaction is especially adaptable to aqueous/biological systems due to (a) the small size of the interaction partners (e.g., compared to streptavidin or antibody-based binding), (b) the range of dissociation constants that can be dialed-in (*K*_d_ ∼ 10^–2^–10^–17^ M), (c) the synthetic ease/scalability of host/guest moieties, and (d) the established bioorthogonality of CB[7]–guest interactions.^49−56^ To employ CB[7] chemistry within a DNA scaffold, straightforward strategies to access oligonucleotides conjugated to CB[7] and guest head-groups are needed.^57−60^ In this work, the CB[7]–DNA conjugates were prepared, by following a report published by our group, wherein monoazido-functionalized CB[7]^61^ is reacted with hexynyl-DNA via copper-catalyzed alkyne–azide click (CuAAC) chemistry.^62^ The synthetic guest molecules attached to the DNA were synthesized by standard NHS-ester/amine coupling reactions. With access to CB[7]- and guest-DNA sequences, we designed a fluorescence-quenching-based reporter assay that probes the CB[7]–guest interaction-driven strand displacement as a function of expulsion of a quenched reporter strand. As shown in Figure 2A, duplex **S:R** is composed of a reporter strand **R** (containing a fluor/quencher pair, 5′-flourescein/3′-dabcyl, hybridized to a complementary sequence **S** that is decorated with a CB[7]-host toehold at its 5′ terminus). **S:R** is fluorescent because the fluorescein/dabcyl pair is separated by a rigid 25 base-pair double helix. However, the addition of input **I** that has the same core sequence as **R** and is attached to a guest moiety at its 3′ end was envisioned to result in HG-TMSD, leading to the release of reporter **R** (which is now quenched due to a random-coil single-stranded state positioning the fluor/quencher pair in proximity), and the concomitant formation of product duplex **S:I**. This latter duplex is thermodynamically stabilized by both host–guest interactions and complementary base-pairing (see Supporting Information, Figure S38).

To determine whether HG-TMSD is operative, we incubated preformed duplex **S:R** (1 μM) for 18 h with 10-fold excess of input sequence **C6-Ad** that contains a high-affinity adamantane guest (*K*_d_ ∼ 10^–10^ M) connected to the DNA backbone via a hexyl linker. As shown in Figure 2B, this input leads to significant quenching of the fluorescein unit (λ_max_ = 518 nm) on **R**, indicating the release of **R** from duplex **S:R**. In particular, 10 equiv of **C6-Ad** results in 65% quenching, while positive control, **R** alone, is 67% quenched. Further, two controls that cannot undergo host–guest interactions were also investigated. The first reaction involves the addition of **C6-Ad** to a duplex that lacks the CB[7] host (S*:R). The second probes the displacement of **S:R** upon addition of an input lacking the adamantane unit (I*). These controls only show minimal fluorescence quenching (15 and 23%). HG-TMSD was confirmed by nondenaturing PAGE (Figure 2B inset), where even 1 equivalent of **C6-Ad** leads to complete disappearance of the substrate **S:R** duplex band along with the emergence of two new bands representing duplex **S:I** and displaced **R**, respectively.

### Fine-Tuning HG-TMSD by Modulating the DNA Domain, Linker Length, and Host–Guest Affinity

The ability to control the dynamics of strand displacement is an important requisite for the development of versatile DNA-based reaction networks. Thus, we investigated the real-time kinetic decay of 1 μM **S:R** with increasing equivalents of **C6-Ad**. For these experiments, the t_0.8_ value (time required to reach 80% signal)^37^ was used to determine kinetic modulation of HG-TMSD. It was found that as the concentration of **C6-Ad** is increased from 1 to 10 equiv, the *t*_0.8_ decreases appreciably from 2.6 ± 0.3 to 0.6 ± 0.1 × 10^3^ s, indicating faster displacement rate for higher equivalents of input (Supporting Information, Figure Si). At 10 equiv of **C6-Ad**, the pseudo-first-order rate constant (*k*) is 1.11 ± 0.07 × 10^–3^ s^–1^. This rate constant is faster than a comparable remote toehold BP-TMSD system (5.29 ± 0.20 × 10^–4^ s^–1^) incorporating a 6 base toehold and contains a synthetic linker connecting the toehold and displacement domains (Supporting Information, Figure S30). However, HG-TMSD is slower than a conventional BP-TMSD system (1.23 ± 0.17 × 10^–2^ s^–1^) with a contiguous DNA domain (Supporting Information, Figure S31i). Another facet of conventional BP-TMSD is that the rate of displacement can be modulated via introduction of mutations. We found that HG-TMSD can also be well-controlled by introducing a singe-base mutation along the DNA domain of input **I**. In particular, as the mutation is introduced closer to the guest toe, the rate decreases appreciably (Supporting Information, Figure S32).

**Figure 3:**
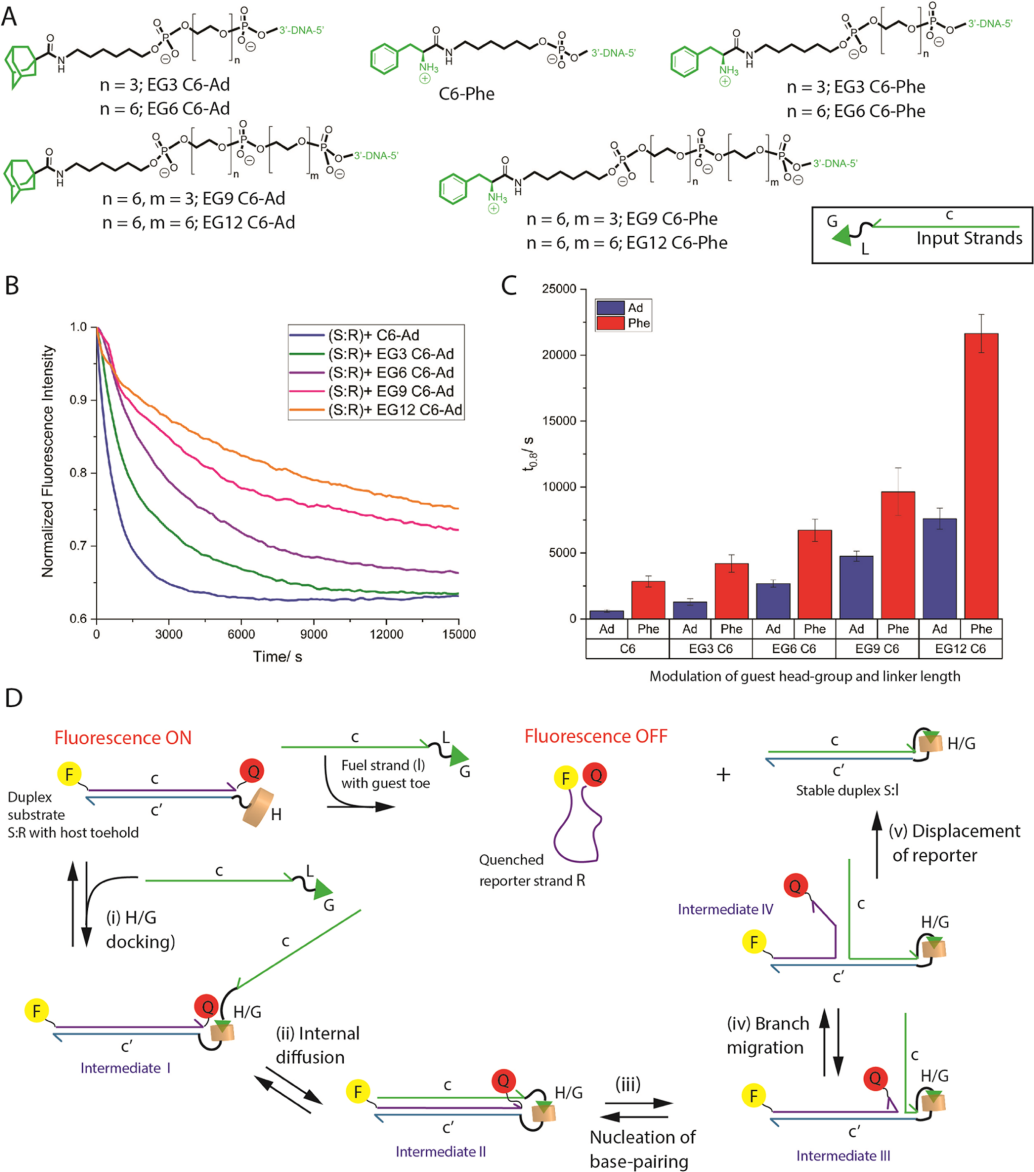
Investigating the effects of guest head-group and linker length on HG-TMSD. (A) Library of guest (adamantane or cationic phenylalanine) tethered input sequences with linker length *L* varied via ethylene glycol spacers. (B) Kinetic decay profile following the fluorescence quenching of the displaced reporter **R** from duplex **S:R** (Figure 3D) as a function of the adamantane-containing input with varying linker length (controls without host–guest interaction are shown in SI Figure Si). (C) A bar-graph depicting the effects of linker length for the adamantane (blue) and phenylalanine (red) tethered inputs on displacement kinetics. (D) Proposed mechanism for HG-TMSD. The first step (i) is the formation of intermediate I via the association of the host and guest units on substrate duplex **S:R** and input strand **I**, respectively. This is followed by (ii) an internal diffusion step where the input **I** explores the volume around the substrate duplex **S:R**, facilitating the alignment of the displacement domain c on **I** with c′ on **S** to (iii) initiate base-pair nucleation. The branch migration step (iv) follows a random-walk process to finally (v) displace the reporter **R** (which is now quenched) and form product **S:I**. Buffer used in these studies: 20 mM Tris, 10 mM NaCl, 5 mM MgCl_2_, pH = 7.5. The results are the mean ± standard deviation (SD) of three independent experiments.

We have also prepared a library of inputs wherein the adamantane guest is separated from the DNA domain via increasing linker length (Figure 3A). In total, five different adamantane–DNA inputs were studied with varying ethylene glycol (EG) units: C6 linker + 0 (**C6-Ad**), 3 (**EG3 C6-Ad**), 6 (**EG6 C6-Ad**), 9 (**EG9 C6-Ad**), or 12 (**EG12 C6-Ad**) EG units. As can be seen from Figure 3B, fine-control over the rate of strand displacement can be achieved by increasing the number of EG units, with the longest linker **EG12 C6-Ad** slowing down the rate 13.5 times compared to **C6-Ad**. To rationalize these results, we modified the remote toehold model developed by Turberfield for BP-TMSD, wherein the traditional base-pair-driven toehold domain is separated from the displacement domain via spacer groups.^37^ In our model (Figure 3D), the first step involves an intermolecular host–guest docking event leading to a ternary complex (**S:R**:**I**, intermediate I). Host–guest binding is followed by internal diffusion leading to intermediate II, where the complementary DNA domains of **I** and **S** are properly positioned to nucleate base-pairing. The nucleation event (intermediate III) will initiate DNA branched migration (occurring through a back and forth random-walk process) leading to eventual displacement of **R** and formation of product duplex **S:I**. While the individual steps are reversible, the inclusion of the high-affinity host–guest interaction is expected to significantly reduce the probability of the stable product duplex **S:I** rebinding to displaced strand **R**. The reason that longer linkers slow down the overall rate of HG-TMSD is likely due to the slowing down of the internal diffusion step, since longer linkers require more time to sample conformations prior to the DNA domains properly aligning for appropriate nucleation of base-pairing.

In addition to changing the linker length, we also attenuated the host–guest interaction using a cationic phenylalanine head-group that has a lower affinity (*K*_d_ ≥ 10^–6^ M)^63^ for CB[7]. A library of phenylalanine-containing inputs was prepared with the varying EG units: C6 linker + 0 (**C6-Phe**), 3 (**EG3 C6-Phe**), 6 (**EG6 C6-Phe**), 9 (**EG9 C6-Phe**), or 12 (**EG12 C6-Phe**) EG units. Interestingly, the phenylalanine-containing inputs exhibited slower HG-TMSD when compared to the corresponding high-affinity adamantane congeners of the same linker length (see Figures 3C and Sii). These observations can be explained by the weaker phenylalanine/CB7 pair having presumably a faster OFF rate (vs. adamantane/CB7 interaction), thereby decreasing the probability that the input strand is attached long enough to the substrate **S** to facilitate productive internal diffusion (step ii), which becomes increasingly slower for linkers with more EG units These results demonstrate that straightforward changes to the input structure enable fine-tuning of HG-TMSD kinetics, with a 36-fold slowdown in rate when **C6-Ad** is replaced by a phenylalanine guest with the longest linker, **EG12 C6-Phe**.

### Competitive Guest-Induced Control of HG-TMSD

**Figure 4:**
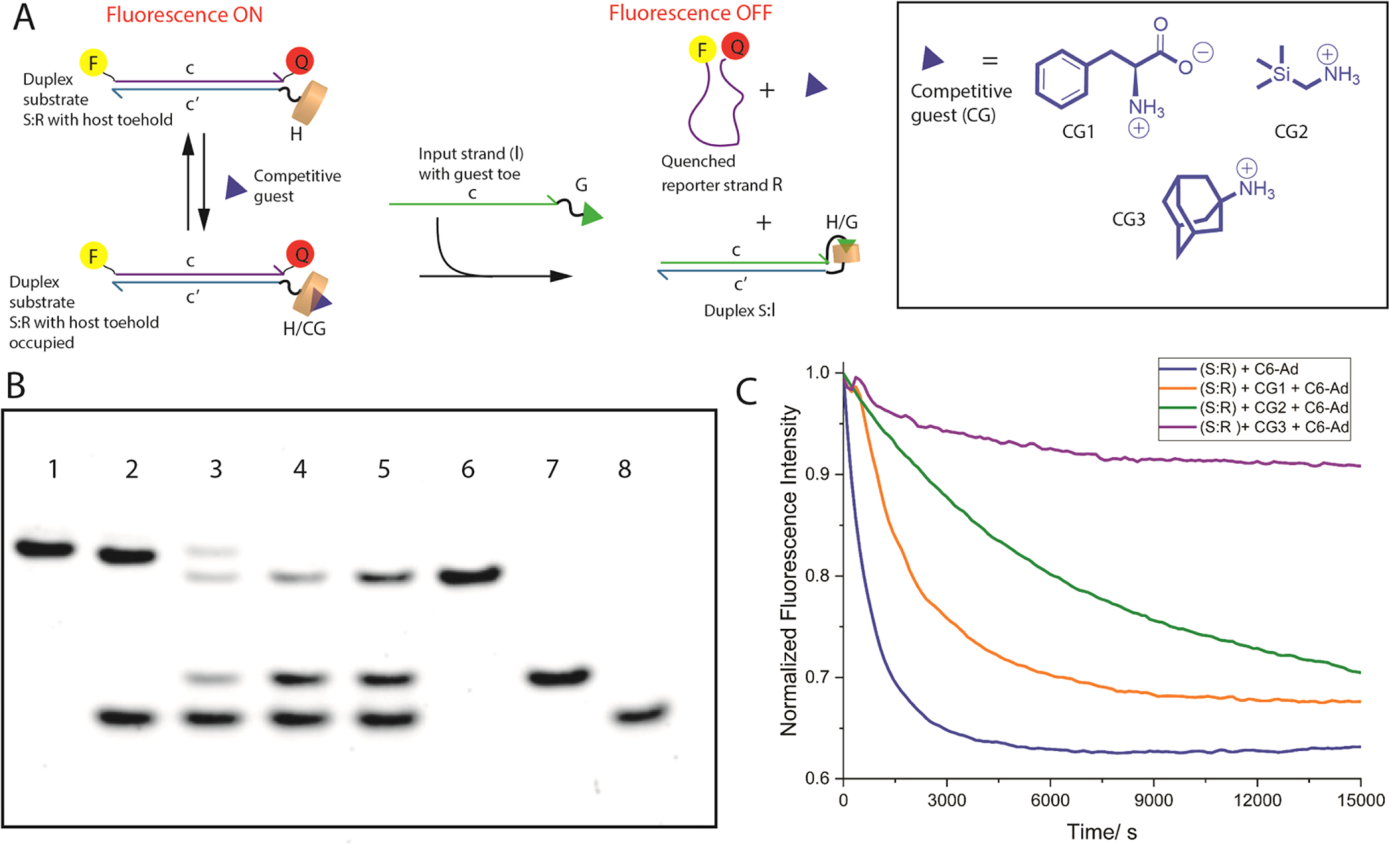
Utilizing competitive small-molecule guests to control HG-TMSD. (A) General scheme illustrating the binding of competitive guests (CGs) into the CB[7] toehold of substrate duplex **S:R** prior to initiation of HG-TMSD by the introduction of **C6-Ad**. The inset shows the structures of the three different CGs used in this study. For these studies, the preformed **S:R** duplex (1 μM) was first incubated with 10 equiv of CGs for 3 h at RT, followed by incubation with 10 equiv of input **C6-Ad** for 10 h at RT. (B) Native PAGE. Lanes: 1 = **S:R** control, 2 = **S:R** + **CG3** + **C6-Ad**, 3 = **S:R** + **CG2** + **C6-Ad**, 4 = **S:R** + **CG1** + **C6-Ad**, 5 = **S:R** + **C6-Ad**, 6 = premade **S:C6-Ad** product control, 7 = **R** only, and 8 = **C6-Ad** only. This study was carried out in TBE buffer at 95 V for 5 h at RT. (C) Kinetic decay profiles for displacement of **R**; **S:R** + **CG3** + **C6-Ad** (violet), **S:R** + **CG2** + **C6-Ad** (green), **S:R** + **CG1** + **C6-Ad** (orange), and **S:R** + **C6-Ad** in the absence of CGs (blue). All samples were prepared in 20 mM Tris, 10 mM NaCl, 5 mM MgCl_2_, pH = 7.5. The results are the mean ± SD of three independent experiments.

Controlled modulation of strand displacement by addition of secondary input molecules that can compete with the toehold domain of the duplex substrate is attractive for the generation of information processing machines (e.g., Boolean logic gates). BP-TMSD is constrained in this regard, since short complementary sequences do not have an appreciable affinity difference compared to the toe domain of the input and hence are not effective modulators (see Supporting Information, Figure S31ii). In contrast, CB[7] can bind to small molecules with a range of affinity, with many (e.g., cationic adamantane derivatives) exhibiting binding that is superior to the neutral adamantane–CB[7] interaction. Hence, we investigated the effects of competitive guests (CGs) on the displacement reaction (Figure 4A). Specifically, three CGs were probed: phenylalanine (**CG1**), (trimethylsilyl)methyl ammonium (**CG2**), and adamantane ammonium (**CG3**) with increasing affinity to CB[7] (∼10^–6^, 10^–9^, and 10^–12^ M, respectively).^64,65^ When PAGE studies were conducted on preformed **S:R** incubated first with CGs, followed by incubation with **C6-Ad** (for 10 h), it was found that HG-TMSD was inversely proportional to the competitive guest–CB[7] affinity (Figure 4B). When **CG3** is used (lane 2), there is no formation of product **S:I** or displaced **R**, indicating that the strong competitor shuts down the reaction. However, the weakest guest **CG1** (lane 4) leads to complete conversion of **S:R** to **S:I**, while incubation with medium-strength **CG2** (lane 3) results in incomplete displacement. Extending the incubation time with **C6-Ad** (to 18 h) for the **CG2** case does allow complete HG-TMSD (Supporting Information, Figure Siii). Using the fluorescence-quenching assay, we found that compared to **C6-Ad** alone, the competitive guest-controlled systems slowed down the reaction by 3.5- and 10-fold, when **CG1** and **CG2** are used, respectively (Figure 4C). In line with PAGE studies, incubation with **CG3** leads to no reaction and the fluorescence quenching does not reach the 80% signal level. These experiments demonstrate that secondary inputs can enable the fine-tuning of HG-TMSD and can even be used for coarse reaction shutdown, thus laying the foundation for complex HG-TMSD-based information processing systems.

### Incorporating HG-TMSD into a DNA Machine to Control Enzyme Activity

**Figure 5:**
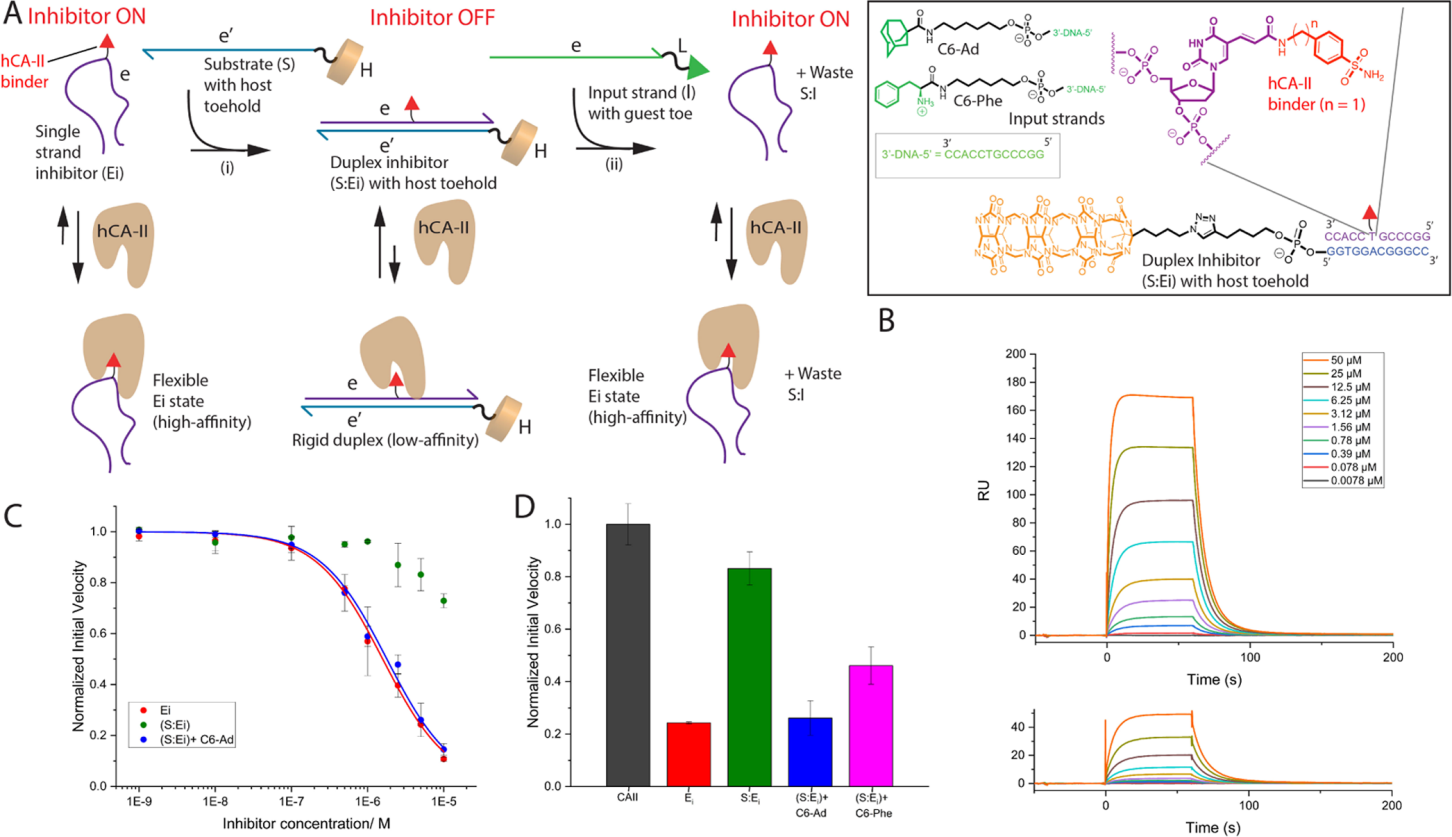
Controlling hCA-II activity via HG-TMSD. (A) Scheme illustrating the regulation of hCA-II activity. Here, the ON state, single strand form of **Ei** (with sequence e and a benzenesulfonamide binder attached to a central dT), binds to the hCA-II active site and inhibits the enzyme. However, upon transition (i) to the rigid duplex form **S:Ei** (OFF state) on addition of a complementary DNA **S** (sequence e′) appended to CB[7], the inhibitor head-group does not bind optimally to the enzyme and therefore the protein remains predominantly active. Incubation (ii) of **S:Ei** with 5 equiv of input **C6-Ad** (sequence e) for 2 h at RT leads to HG-TMSD and the release of **Ei**, reverting to its ON state. Inset: DNA sequences used for this portion of the study. The small-molecule inhibitor is shown in red. (B) Surface plasmon resonance (SPR) sensograms; top panel: **Ei** binding to hCA-II; bottom panel: **S:Ei** duplex binding to hCA-II. A 0.5% surfactant P20 buffer at pH 7.4 was used as the running buffer. Binding measurements were obtained by flowing oligonucleotide strands at increasing concentrations (7.8 x 10^–3^ to 50 μM) over immobilized hCA-II protein on CM5 chips containing carboxymethylated dextran (Cytiva). (C) Normalized hCA-II activity (following the conversion of *p*-nitrophenyl acetate to *p*-nitrophenol) upon addition of **Ei** (red profile), **S:Ei** duplex (green), and **S:Ei** + 5 equiv of **C6-Ad** (blue). The results are the mean ± SD of three independent experiments. The data were fit to a competitive inhibition model. (D) Normalized hCA-II activity at 5 μM concentration of **Ei** (red), **S:Ei** duplex (green), **S:Ei** + 5 equiv of **C6-Ad** (blue), **S:Ei** + 5 equiv of **C6-Phe** (pink), and hCA-II only (black). Controls without host–guest interaction are shown in SI Figure S34. All samples were prepared in 20 mM Tris, 10 mM NaCl, 5 mM MgCl_2_, pH = 7.5. The results are the mean ± SD of three independent experiments.

After establishing the versatility of our supramolecular approach to strand displacement, we wanted to highlight how HG-TMSD can be seamlessly integrated with traditional dynamic DNA nanotechnology schemes to achieve specific function. Our group (and others) is interested in using supramolecular chemistry, in combination with DNA, to control protein activity.^66−71^ Herein, we demonstrate that HG-TMSD can be used to toggle a DNA–sulfonamide conjugate, an inhibitor of human carbonic anhydrase-II (hCA-II) from an active ON state, wherein the enzyme is inhibited, to a rigid duplex-based OFF state. Our design features a single-stranded DNA–protein inhibitor conjugate (**Ei**) decorated with a benzenesulfonamide head-group on an internal dT residue (Figure 5A). In the single-stranded state, **Ei** is flexible and should provide minimal steric hindrance to the protein-binding head-group and thus should effectively inhibit hCA-II (ON state of **Ei**). The addition of a complementary substrate strand **S** tethered to CB[7] will lead to a rigid duplex **S:Ei** that will decrease the ability of the inhibitor head-group from binding optimally to hCA-II (due to duplex sterics) resulting in an OFF state. However, the introduction of a guest containing input **I** will result in HG-TMSD that reverts the **S:Ei** duplex to single-stranded **Ei** (and waste duplex **S:I**), thereby regaining its inhibition capacity.

We performed surface plasmon resonance (SPR) experiments to interrogate the differential binding abilities of **Ei** and **S:Ei** (Figure 5B). Interestingly, the single-stranded **Ei** (with a methylene spacer between the sulfonamide and dT residue, *n* = 1) exhibited a *K*_d_ of 12.04 × 10^–6^ M, whereas *K*_d_ for the duplex-state **S:Ei** was found to be 44.55 × 10^–6^ M. Note: the length of the linker connecting the sulfonamide is critical, since a congener with a longer linker (*n* = 2) does not show such differential binding (Supporting Information, Figure S33i). Furthermore, when hCA-II esterase activity assays were performed on **Ei**, an inhibition constant *K*_i_ = 1.27 ± 0.04 × 10^–6^ M was obtained (Figure 5C). In contrast, the **S:Ei** duplex did not reach 50% inhibition even at 1.0 × 10^–5^ M (where the titration was halted due to the need for high duplex concentrations). Importantly, when deactivated **S:Ei** was incubated with **C6-Ad** (5 equiv) and then introduced to the enzyme, we observed a *K*_i_ of 1.43 ± 0.11 × 10^–6^ M, demonstrating reactivation of **Ei**. To better describe the inhibition, fixed concentration (5.0 × 10^–6^ M) of various inhibitor species was probed (Figure 5D). Here, **Ei** alone significantly drops the enzyme activity to 24%, while the enzyme activity is 83% in the presence of **S:Ei**. Incubation of **S:Ei** with **C6-Ad** leads to reduced hCA-II activity (26%). However, control systems lacking the CB[7] (Supporting Information, Figure S34, light blue bar) or adamantane (pink bar) partners do not significantly reduce the enzyme activity due to lack of HG-TMSD. Moreover, when an input with a weaker binding phenylalanine head-group is used (**C6-Phe**), the displacement of **Ei** from the **S:Ei** duplex does not go to completion and 46% hCA-II activity is observed. These experiments not only demonstrate that HG-TMSD can be used to develop dynamic machines with enzyme inhibition activity but also that dialing-in the host–guest interaction strength can lead to fine-control of enzyme activity levels at a given inhibitor concentration.

### Design of a Relay Composed of BP-TMSD and HG-TMSD to Detect Tumor-Associated microRNA

**Figure 6:**
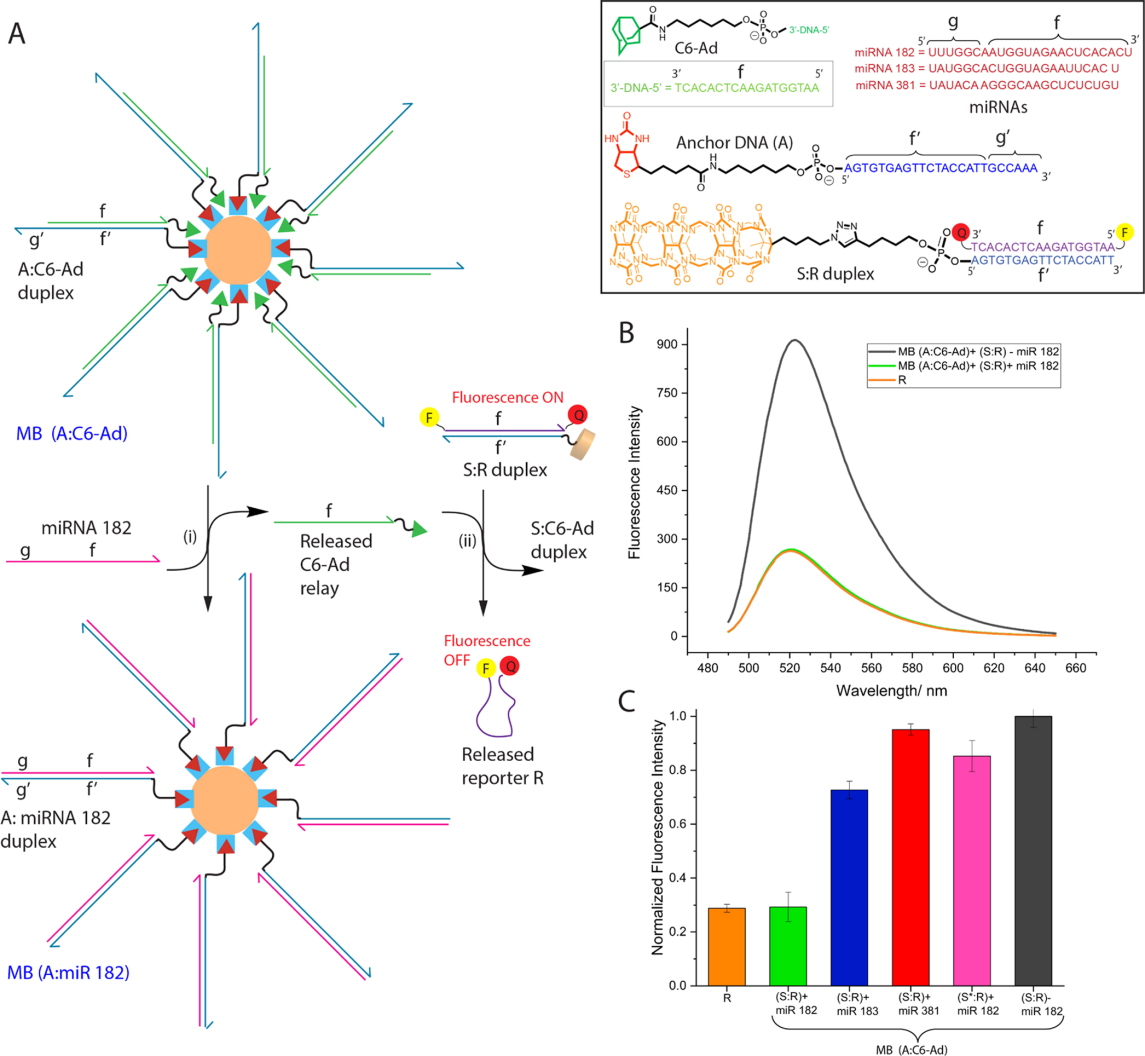
Detection of miR-182 via a layered reaction featuring HG-TMSD. (A) Cascade reaction using tandem BP-TMSD and HG-TMSD to detect **miR-182**. An anchor DNA strand **A** (with sequence f′ and canonical BP-toehold g′) attached to magnetic beads MB (via biotin–streptavidin interactions) is hybridized with a **C6-Ad** relay (sequence f). Upon (i) introduction of **miR-182** (which contains toe sequence g and is fully complementary to **A**), BP-TMSD occurs resulting in the displacement of relay **C6-Ad**. In the presence of the **S:R** duplex, the displaced **C6-Ad** initiates HG-TMSD releasing reporter **R** in a fluorescence-quenched state. Inset: Structures and sequences used in this portion of the study. (B) The displacement of reporter **R** is monitored by the quenching of fluorescence (green profile). Also shown is the high fluorescence of **S:R** in the absence of **miR-182** (black), and the quenched fluorescence of control **R** only (orange). (C) Normalized fluorescence emission at 522 nm (excitation 470 nm) after 1 h incubation (at RT) of **MB (A:C6-Ad)** with **S:R** + **miR-182** (green), **S:R** + **miR-183** (blue), **S:R** + **miR-381** (red), **S:R** – **miR-182** (black), and S*:R + **miR-182** (pink). The positive control, **R** alone, is shown in orange. The buffer used in the binding/displacement studies was 20 mM Tris, 10 mM NaCl, 5 mM MgCl_2_, pH = 7.5. The results are the mean ± SD of three independent experiments.

In an effort to further demonstrate the utility of HG-TMSD, we designed a microRNA (miR) detection system that combines (a) BP-TMSD and HG-TMSD, (b) a **C6-Ad** relay sequence fastened to magnetic beads, and (c) the fluorescence-quenching reporter assay developed above. This layered reaction enables the selective detection of **miR-182**, a member of the miR-183 cluster family, which is overexpressed in multiple cancers.^72^ Interestingly, **miR-182** is present in exosomes isolated from human serum and thus is an attractive target for diagnostic applications.^73^ Our reaction scheme (Figure 6A) is based on magnetic beads fastened (via biotin–streptavidin interactions) to duplex DNA: **MB** (**A:C6-Ad**). The duplex is composed of **C6-Ad** hybridized to anchor DNA **A**. The reaction is initiated by the binding of **miR-182** to the 6 base-pair toehold (g′) of anchor **A**. This results in BP-TMSD that releases the relay **C6-Ad** into solution and forms waste duplex composed of **miR-182** and anchor **A** on the magnetic bead scaffold **MB** (**A:miR-182**). The released **C6-Ad** is an input for an *in situ* downstream reaction wherein HG-TMSD is used to trigger the formation of fluorescence-quenched **R**. Upon magnetic separation of the waste duplex **MB** (**A:miR-182**), the extent of fluorescence quenching can be readily followed.

Before initiation with **miR-182**, all sequences are in a stable duplex form and importantly Watson–Crick–Franklin and host–guest overhangs are orthogonal and thus do not cross-react. Further, to minimize potential host–guest interactions between bead-bound **C6-Ad** and the **S:R** duplex, the adamantane head-group on **MB** (**A:C6-Ad**) is positioned toward the magnetic bead surface. Indeed, in the absence of **miR-182**, the system shows no fluorescence quenching. In marked contrast, the fluorescence quenching is significant upon introduction of 10 equiv (vs. **S:R** duplex) of **miR-182**, which is similar to the level of quenching observed for control **R** alone (Figure 6B). To probe the selectivity of this system toward **miR-182**, two other miR sequences were interrogated. The first, **miR-183**, belonging to the same family as of **miR-182** and has high sequence homology (four mismatches vs **miR-182**). The second, **miR-381**, has 13 mismatches. The normalized fluorescence intensity for these experiments is shown in Figure 6C. Here, **miR-183** and **miR-381** exhibit 27 and 5% quenching. However, **miR-182** leads to 71% quenching, similar to **R** alone. To ensure that HG-TMSD is needed to facilitate this layered reaction, we performed control experiments with duplex S*:R that does not contain the CB[7] host (see Figure 6C, pink bar). This control shows high fluorescence even after addition of **miR-182**, indicating the importance of host–guest interactions in the detection scheme. Using this sensing strategy, the limit of detection for **miR-182** was found to be 6.3 pmol (see Supporting Information, Figure S37).

## Conclusions

We have disclosed the design and development of a novel isothermal DNA strand-displacement mechanism that is initiated by synthetic supramolecular chemistry. HG-TMSD system is attractive since it harnesses CB[7] host–guest interactions that are small in size and orthogonal to DNA base-pairing (bioorthogonal, in general). Further, the interplay of host–guest affinity and linker length can be used to fine-tune the rate of HG-TMSD. In contrast to conventional strand displacement, HG-TMSD can be precisely modulated by the introduction of competing small-molecule guests (that can bind to the CB[7] toehold domain with a range of affinity constants). In addition to developing a fluorescence-based reporter assay to show HG-TMSD activity and modulation, we have also integrated HG-TMSD into functional DNA-based systems. In particular, this work describes a HG-TMSD-based DNA machine that regulates enzyme activity via toggling of an inhibitor between single-stranded and duplex states. Furthermore, we detail a tandem reaction using BP-TMSD, HG-TMSD, and the developed fluorescence assay to selectively detect **miR-182**, an important cancer biomarker. In sum, this work introduces a highly versatile supramolecular approach to DNA strand displacement that is envisioned to broadly expand the toolbox of dynamic DNA chemistry and nanotechnology. While this strategy neither currently has the same level of programmability nor is it as fast as conventional strand displacement but it expands the toolbox of DNA nanotechnology. Further, this is an important step toward fully synthetic “strand” displacement systems (e.g., where the core DNA domain is also replaced with a synthetic molecular recognition congener).

## Supporting Information Available

All relevant experimental procedures including detailed synthesis and characterization of synthetically functionalized DNA strands (MALDI-TOF, RP-HPLC) (PDF)
