# Supplementary material for: DNA Strand Displacement Driven by Host–Guest Interactions
Source: J Am Chem Soc. 2022 Sep 5;144(36):16502–11. doi: 10.1021/jacs.2c05726 (PMC9479067; doi:10.1021/jacs.2c05726)
Supplement: Supplementary file 1 — ja2c05726_si_001.pdf [file ja2c05726_si_001.pdf]

Supplementary Information

DNA Strand Displacement Driven by Host-Guest Interactions

Dilanka V. D. Walpita Kankanamalage,[1] Jennifer H.T. Tran,[2] Noah Beltrami,[1] Kun Meng, [1] Xiao Zhou,[1] Pravin Pathak,[1] Lyle Isaacs,[3] Alexander L. Burin, [1] Mehnaaz F. Ali,[2] * Janarthanan Jayawickramarajah[1] *

1. Department of Chemistry, Tulane University, 2015 Percival Stern Hall, New Orleans, Louisiana 70118, United States
2. Department of Chemistry, Xavier University of Louisiana, 1 Drexel Drive, New Orleans, Louisiana 70125, United States
3. Department of Chemistry and Biochemistry, University of Maryland, College Park, Maryland 20742, United States

Table of Contents

S1. Materials and Methods

- S1.1. Materials
- S1.2. General Instrumentation
- S1.3. Key Experimental Procedures
- S1.4. Key Experimental Figures

S2. Synthesis of DNA conjugates

- S2.1. Synthesis of CB[7] conjugated DNA
- S2.2. Synthesis of Adamantane conjugated DNA
- S2.3. Synthesis of Phenylalanine conjugated DNA
- S2.4. Synthesis of 4-(aminomethyl) benzenesulfonamide and 4-(aminoethyl) benzenesulfonamide conjugated DNA
- S2.5. RP-HPLC purification of DNA conjugates
- S2.6. MALDI-TOF MS analysis of DNA conjugates

S3. Characterization of Small Molecule-DNA Conjugates

- S3.1. Characterization of DNA conjugates used in the development of HG-TMSD
- S3.2. Characterization of DNA conjugates used in DNA nanomachines that utilize HG-TMSD to control hCA-II activity
- S3.3. Characterization of DNA conjugates used in sequential BP-TMSD and HG-TMSD reactions to detect microRNA-182

S4. Supporting Figures

- S4.1. Comparison of HG-TMSD displacement kinetics with conventional BP-TMSD
- S4.2. HG-TMSD mutation studies
- S4.3. Supplementary figures relevant to hCA-II inhibition
- S4.4. Supplementary figures relevant to miR-182 detection

S5. Supplementary Tables

- S5.1. DNA sequences used in the development of HG-TMSD
- S5.2. DNA sequences used in the DNA nanomachines that utilize HG-TMSD to control hCA-II activity
- S5.3. DNA sequences used in the sequential BP-TMSD and HG-TMSD reactions to detect miR-182
- S5.4. Input DNA sequences used in HG-TMSD mutation studies
- S5.5. DNA sequences used in thermal denaturation studies
- S5.6. RP-HPLC eluent gradient

S6. Thermal Denaturation Studies of Host-Guest Stabilized DNA Duplexes

- S6.1. Synthesis and characterization of DNA-small molecule conjugates
- S6.2. General experimental of thermal denaturation studies
- S6.2. Results and discussion of thermal denaturation studies of host/ guest stabilized DNA duplexes.

S7. References

S1. Materials and Methods

S1.1. Materials

Unless otherwise noted, all chemicals and solvents were purchased from Sigma Aldrich or Fisher Scientific and used without further purification. Small molecule competitive guest, Trimethylsilylmethylamine (**CG2**) was purchased from AA blocks LLC. Adamantane-NHS ester was synthesized according to a previously reported literature protocol.[1] Monofunctionalized CB[7]-N3 was synthesized via a reported procedure.[2] The core DNA sequences used to construct the host and guest functionalized single-stranded DNA systems were prepared by the W. M. Keck Foundation Biotechnology Resource Laboratory (located at Yale University) using standard automated solid phase synthesis. In addition to standard nucleoside phosphoramidites, the required modified phosphoramidites (e.g., 5' hexynyl phosphoramidite, 6-fluorescein serinol phosphoramidite, 3' dabcyl CPG, 3' PT amino modifier C6 CPG, spacer phosphoramidite 9, spacer phosphoramidite 18, spacer C12 CE phosphoramidite, NHS ester carboxy dT, 5' biotin phosphoramidite, and 3' fluorescein CPG) were purchased from Glen Research. The DNA sequences were designed with the help of m-fold and OligoEvaluator™ (Sigma Aldrich) in order to minimize unwanted secondary structures.[3]

S1.2. General Instrumentation

All RP-HPLC purifications were obtained using a Varian Prostar HPLC system, equipped with an Agilent PLRP-S 100 Å 5 µm 4.6 × 250 mm reverse phase column. MALDI-TOF MS characterization was acquired using a Bruker Autoflex III matrix-assisted laser desorption time-of-flight mass spectrometer (MALDI-TOF MS) or a Bruker Daltonics Autoflex Speed MALDI TOF/TOF, with positive ion and linear detection modes. The concentration of purified DNA conjugates was quantified on a HP 8452A Diode Array Spectrophotometer, based on their absorption (at 260 nm) using nearest neighbor calculations. NMR characterization of adamantane-NHS ester was recorded on a Bruker 300 MHz spectrometer. Native PAGE experiments were performed in an Invitrogen Xcell Sure Lock Mini-Cell electrophoresis device, and the gels were imaged using a BioRad 2.2.0.8 / ChemiDoc Imaging System with Image Lab Software. All fluorescence quenching studies relevant to the development of HG-TMSD, and colorimetric assays for hCA-II activity were performed on a PerkinElmer EnSpire Multimode Plate Reader whereas the fluorescence studies relevant to the miR-182 detection were conducted on a SpectraMax M3 Multi-Mode Microplate Reader. SPR experiments pertinent to the binding of synthetic DNA to hCA-II was performed on a Biacore T200.

S1.3. Key Experimental Procedures

All incubations were done at room temperature unless otherwise stated.

Development of host-guest based toehold mediated strand displacement

Native PAGE. Nondenaturing polyacrylamide gel electrophoresis was conducted on an Invitrogen Xcell SureLock Mini-Cell electrophoresis device. Precast Novex^{TM} TBE gels (20%) were purchased from Thermofisher Scientific. A 20 mM Tris buffer containing 10 mM NaCl and 5 mM $MgCl_2$, pH = 7.5, was used for all sample preparations. For the HG-TMSD assays, 1 µM of **S:R** duplex was prepared by mixing equal parts of **S** and **R** sequences, followed by heating to 65 °C for 3 min and slowly cooling down to RT for 3 hours. A solution of 0.1 nmol **S:R** duplex was then incubated with 10 eq. of fuel strand at RT for 18 hours prior to native PAGE. For all gel studies with competing guests, 0.1 nmol **S:R** duplex was first incubated with 10 eq. of **CG1**, **CG2** and **CG3** separately for 3 hours at RT, followed by addition of 10 eq. of **C6-Ad** and incubating at RT for 10 or 18 hours. Loading samples were prepared by mixing 10 µL of final DNA solution with 1.5 µL loading dye (DNA gel loading dye 6x, obtained from Thermofisher Scientific). All PAGE experiments were carried out at RT for 5 hours under constant voltage (95 V) with 1x TBE (89 mM Tris, 89 mM boric acid, 2 mM EDTA) running buffer. SYBR safe DNA gel stain in 0.5x TBE (obtained from Thermofisher as ready-to-use solution) was used to stain the gels at RT for 20 min and imaged using a BioRad 2.2.0.8 / ChemiDoc Imaging System with Image Lab Software.

Fluorescence-Quenching Reporter Assay. Fluorescence studies were carried out using a PerkinElmer EnSpire Multimode Plate Reader using Costar- 96 well standard black plates.

(a) ***Steady-State Experiments.*** The **S:R** duplex (0.1 nmol), prepared as mentioned above, was incubated with input strands (1 nmol) at RT for 18 hours followed by measurement of the fluorescence. Excitation wavelength was 470 nm, and fluorescence emission was collected at nanometer increments with a spectral maximum emission observed at 518 nm.

(b) ***Displacement Kinetics.*** For the kinetic studies, the fluorescence-quenching (at 518 nm) was monitored immediately upon addition of respective fuel strands (1 nmol) to a solution containing **S:R** duplex (0.1 nmol). Data was recorded at 120s time interval for a period of 1.5×10^4 s and 3.0×10^4 s when Ad-DNA and Phe-DNA were used as input strands, respectively. For the kinetic assays involving competing small molecule guests, 1 nmol of **CG1**, **CG2**, or **CG3** were first incubated with **S:R** duplex (0.1 nmol) and gently agitated in a mechanical shaker for 3 hours at RT prior to addition of **C6-Ad** input strand (1 nmol).

The kinetic data were normalized (dividing by fluorescence value at t=0 for each well), and the normalized fluorescence intensity was plotted against time to generate kinetic decay profiles (using OriginPro 2019 software). The normalized kinetic decay profiles were smoothed using the Savitsky-Golay method (points of window: 5, polynomial order: 2).

DNA machine that utilizes HG-TMSD to control hCA-II activity

Surface Plasmon Resonance Binding Assay. Human recombinant hCA-II expressed in E. coli, buffered aqueous solution) was purchased from Sigma-Aldrich. The binding of synthetic DNA to hCA-II was performed on a Biacore T200 SPR instrument. Series S CM5 chips containing carboxymethylated dextran (Cytiva) were used for all experiments. The hCA-II protein was immobilized using amine-coupling chemistry at room temperature. PBS-P+ (pH 7.4, containing 0.5% surfactant P20) buffer was used as the running buffer. Prior to protein immobilization, the detector was normalized using BIA normalizing solution (70% w/w glycerol solution, Cytiva). Using a flow rate of 10 µL/min, the chip surface was activated with a 7 min injection of 0.4 M EDC and 0.1 M NHS, followed by a 7 min injection of 100 µg/mL hCA-II protein in 10 mM sodium acetate solution (pH 5.0). Unreacted NHS groups were blocked with a 7 min injection of 1.0 M ethanolamine. Using this procedure, hCA-II was immobilized on the chip to 4,253 response units. The reference flow cell was left unmodified. All synthetic DNA systems used were diluted from 2.00 µM to 0.06 µM in running buffer and injected for 60 seconds at a flow rate of 30 µL/min and dissociation was monitored for 5 min. As a regeneration step, each cycle of analyte injection was followed by a 1 min injection of running buffer at a flow rate of 30 µL/min. Data sets were blank correlated and analyzed with the Biacore T200 evaluation software using a 1:1 binding model.

hCA-II Esterase Activity Inhibition Assay. The enzyme inhibition assay was conducted by dissolving hCA-II (1×10^{-7} M) in assay buffer (20 mM Tris, 10 mM NaCl, 5 mM $MgCl_2$, pH 7.5) and incubating in a 96-well plate with a dilution series of inhibitors for 1 hour at RT. A concentration series (1.0×10^{-9} to 1.0×10^{-5} M) of single stranded **Ei** or duplex **S:Ei** were used as inhibitors. The colorimetric assay was initiated by adding substrate *p*-nitrophenylactetate (*p*-NPA, 1 mM), and the UV-vis absorption change at 405 nm was recorded (which corresponds to the production of *p*-nitrophenolate) by using a PerkinElmer EnSpire Multimode Plate Reader. For the inhibition experiment involving the displaced **Ei**, 1 eq. of **S:Ei** duplex was incubated with 5 eq. of **C6-Ad** for 2 hours at RT. From this stock solution, a dilution series was prepared (conc. 1.0×10^{-9} M - 1.0×10^{-5} M) for subsequent enzyme inhibition. Normalization of the initial reaction velocity was performed by assigning a maximum value of 1 to the initial reaction velocity obtained from the hCA-II activity in the absence of any inhibitor for each series of experiments. Specific initial reaction velocities in the presence of inhibitors in these studies were converted to proportions of this value (ranging from 0 to 1). In order to estimate the K_i (the inhibition constant of the inhibitor), the normalized initial reaction velocity (V) was plotted against the corresponding inhibitor concentration (I), and the K_i value was obtained by fitting, via nonlinear regression, to equation 1, using OriginPro 2019 software[4].

$$V = \frac{K_m + [S]}{[S] + K_m\left(1 + \frac{[I]}{K_i}\right)} \qquad (1)$$

Here, [S] is the total concentration of the substrate *p*-NPA (1.0 mM). The Michaelis−Menten constant K_m (the substrate concentration that gives a reaction rate equal to one-half the maximum rate) was first obtained for *p*-NPA hydrolysis catalyzed by hCA-II in the absence of inhibitors by fitting the data (Supplementary Figure 35) to the Michaelis−Menten equation (2) where V_0 is the initial velocity and V_{max} is the maximum velocity of the reaction. The K_m value was calculated to be 4.4 ± 0.5 mM, and V_{max} was 3.97± 0.14 x10^{-3} O.D./min.

$$V_0 = \frac{V_{max}[S]}{K_m + [S]} \qquad (2)$$

Sequential BP-TMSD and HG-TMSD reactions to detect microRNA-182

a) *Preparation of magnetic beads.* Pierce streptavidin magnetic beads **(MB)** with 1 µm mean diameter and binding capacity of 3.5 nmol/mg towards biotin was purchased from Thermo Fisher Scientific (bead concentration: 1 mg/100 µL). For each experiment, a 100 µL homogenous bead solution was transferred to an eppendorf tube and the supernatant was discarded using a magnetic separator. The captured beads were rinsed once with 400 µL of 0.01 M PBS buffer (pH 7.4) and twice with 400 µL of assay buffer (20 mM Tris, 10 mM NaCl, 5 mM $MgCl_2$, pH 7.5).

b) *Loading of duplex **A:C6-Ad** on to **MB**.* The duplex **A:C6-Ad** was prepared by hybridizing 100 µL of 20 µM anchor DNA strand **A** carrying a 5'-biotin modification, with 100 µL of 24 µM **C6-Ad**. Duplex **A:C6-Ad** (200 µL in assay buffer/ 2.0 nmol) was then added to the **MB**, incubated, and gently agitated in a mechanical shaker for 3 hours at RT. The supernatant was discarded, and the beads were thoroughly rinsed three times with 400 µL of assay buffer to ensure no residual unbound **A:C6-Ad** duplex in solution (following DNA absorption at 260 nm using a Nanodrop, Thermofisher 2000/2000c).

c) *Determination of **MB** bound duplex **A:C6-Ad**.* In parallel set of experiments, an anchor DNA strand **A'** carrying 5' -biotin and 3'-fluorescein modifications, was hybridized with **C6-Ad** in 1:1.2 molar ratio to obtain duplex **A':C6-Ad**. Fluorescence was used to estimate the loading (1.58 nmol) of this anchor duplex onto the **MB.** (See Supplementary Figure 36 for details). It was assumed that the loading of the non-fluorescent duplex **A:C6-Ad** (incubated with **MB** under identical experimental conditions, *supra*), was also 1.58 nmol.

d) *Selective detection of **miR-182**.* To initiate the sequential reaction, 0.1 nmol of **S:R** duplex (in 100 µL assay buffer) was added to the rinsed beads followed by the addition of 1.0 nmol of **miR-182** (in 100 µL assay buffer), and incubated at RT for 1 hour, at which point the MB was magnetically separated, and the fluorescence of the solution analyzed. In order to probe the selectivity of the system, the same protocol was followed using two other miR sequences: **miR-183** and **miR-381**. When **S*** (control DNA

without the host-CB[7] modification) was investigated, 0.1 nmol **S*:R** duplex was incubated with 1.0 nmol **miR-182** and the same protocol was followed.

For the miR-detection system, the fluorescence intensity was monitored against wavelength on a SpectraMax M3 Multi-Mode Microplate Reader using Costar- 96 well standard black plates. An excitation wavelength of 470 nm was used, and emission data was collected at each nanometer with a spectral maximum observed at 522 nm.

S1.4. Key Experimental Figures

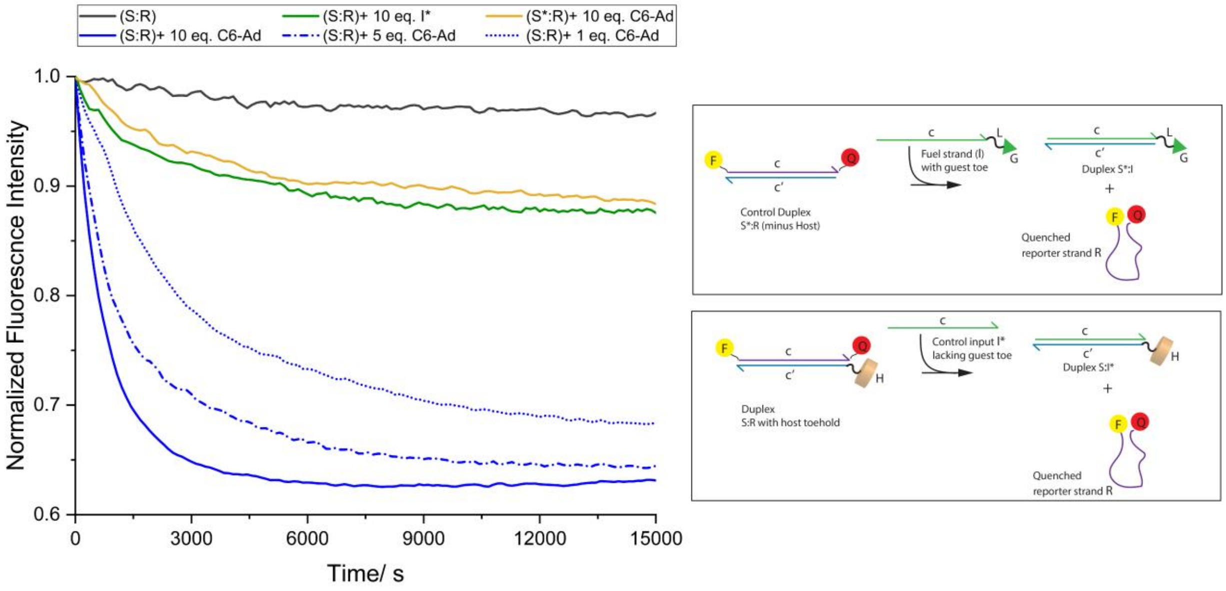

Supplementary Figure i. Kinetic decay profiles with increasing equivalents of C6-Ad and negative controls lacking H/G interactions. (i) Negative controls; **S:R** duplex alone (black profile), **S:R** duplex with fuel strand lacking the guest head-group: **S:R+ I*** (green profile), **S:R** duplex lacking CB7 host; **S*:R + C6-Ad** (yellow profile), (ii) Increasing the rate of displacement using higher equivalents of **C6-Ad** fuel strand; **S:R** + 1 equivalent of **C6-Ad** (blue dotted line)/ 5 equivalents of **C6-Ad** (blue dashed line)/ 10 equivalent of **C6-Ad** (blue solid line). Increasing equivalents (1/5/10) of **C6-Ad** resulted in $t_{0.8}$ of 2.60 ± 0.34 x 10^3 s, 0.96 ± 0.24 x 10^3 s, and 0.60 ± 0.12 x 10^3 s, respectively. The buffer used in the displacement studies was: 20 mM Tris, 10 mM NaCl, 5 mM $MgCl_2$, pH = 7.5. Results are the mean of 3 independent experiments. **Inset:** Schematic for the control systems lacking the host-guest interactions.

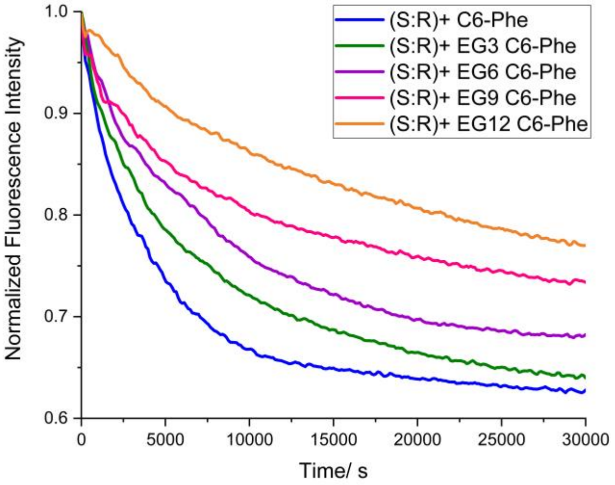

Supplementary Figure ii. Kinetic decay profiles for S:R displacement by phenylalanine headgroup containing fuel strand library. **S:R** displacement by; **C6-Phe** (blue profile), **EG3 C6-Phe** (green profile), **EG6 C6-Phe** (violet profile), **EG9 C6-Phe** (pink profile), **EG12 C6-Phe** (orange profile). Increasing linker length affords longer $t_{0.8}$ values implying slower displacement kinetics. Since the phenylalainine guest slows down HG-TMSD (compared to the adamantane guest), the kinetic experiments were carried out for 3.0 x 10^4 seconds (twice as long for the adamantane counterpart). The buffer used in the displacement studies was: 20 mM Tris, 10 mM NaCl, 5 mM $MgCl_2$, pH = 7.5. Results are the mean of 3 independent experiments.

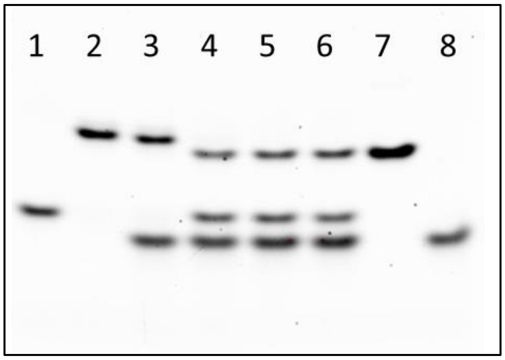

Supplementary Figure iii. Native PAGE to monitor competitive HG-TMSD at 18 hr incubation with C6-Ad. For these studies, to the preformed **S:R** duplex (1 µM) was first incubated with 10 eq. of CGs for 3 hr, followed by incubation with 10 eq. of input **C6-Ad** for 18 hr. **(B)** Native PAGE Lanes; 1 = **R** only, 2 = **S:R** control, 3 = **S:R** + **CG3** + **C6-Ad**, 4 = **S:R** + **CG2** + **C6-Ad**, 5 = **S:R** + **CG1** + **C6-Ad**, 6 = **S:R** + **C6-Ad**, 7 = premade **S:C6-Ad** product control, 8 = **C6-Ad** only. PAGE was carried out in 10% TBE buffer at 95 V for 5 hr at RT. This study confirmed that **C6-Ad** is capable of completely transforming **S:R** duplex into product duplex in the presence of CG2 at longer incubation period (compared to main text Figure 4B).

S2. Synthesis of DNA Conjugates

S2.1. Synthesis of CB[7]-conjugated DNA

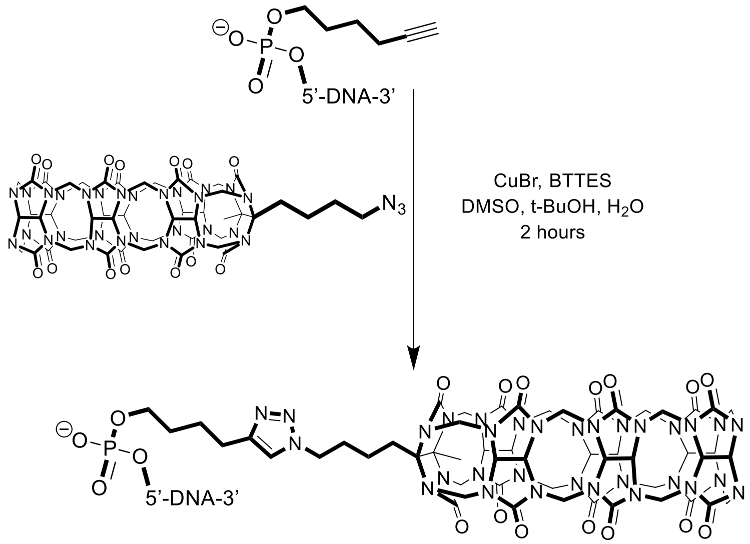

Supplementary Figure 1. Synthesis scheme of CB[7]-conjugated DNA.

A solution of 1 mg (0.8 µmol) CB[7]-N3 in 100 µL H_2O was added to an eppendorf tube containing 0.1 µmol 5' hexynyl modified DNA in 100 µL H_2O. In a separate vial, 15 µL CuBr solution (100 mM in DMSO/tBuOH 3:1) and 60 µL 3-(4-((Bis((1-(*tert*-butyl)-1*H*-1,2,3-triazol-4-yl)methyl)amino)methyl)-1*H*-1,2,3-triazol-1-yl)propane-1-sulfonic acid; (BTTES) ligand solution (100 mM in DMSO/tBuOH 3:1) were vortexed and then added to the eppendorf tube containing DNA + CB[7]-N3 solution. The resulting solution was shaken at room temperature for 2 hours. A G25 micro spin column was used to desalt the solution, and then the crude product was purified by RP-HPLC. The mass of the CB[7]-DNA conjugate was confirmed with MALDI-TOF.

This general protocol was followed for the synthesis, purification, and characterization of all CB[7] labeled **S** strands used in this study. See Supplementary Figure 6, 21, and 26 for their characterization details. See Supplementary Tables 1-3 for individual sequences.

S2.2. Synthesis of Adamantane-conjugated DNA

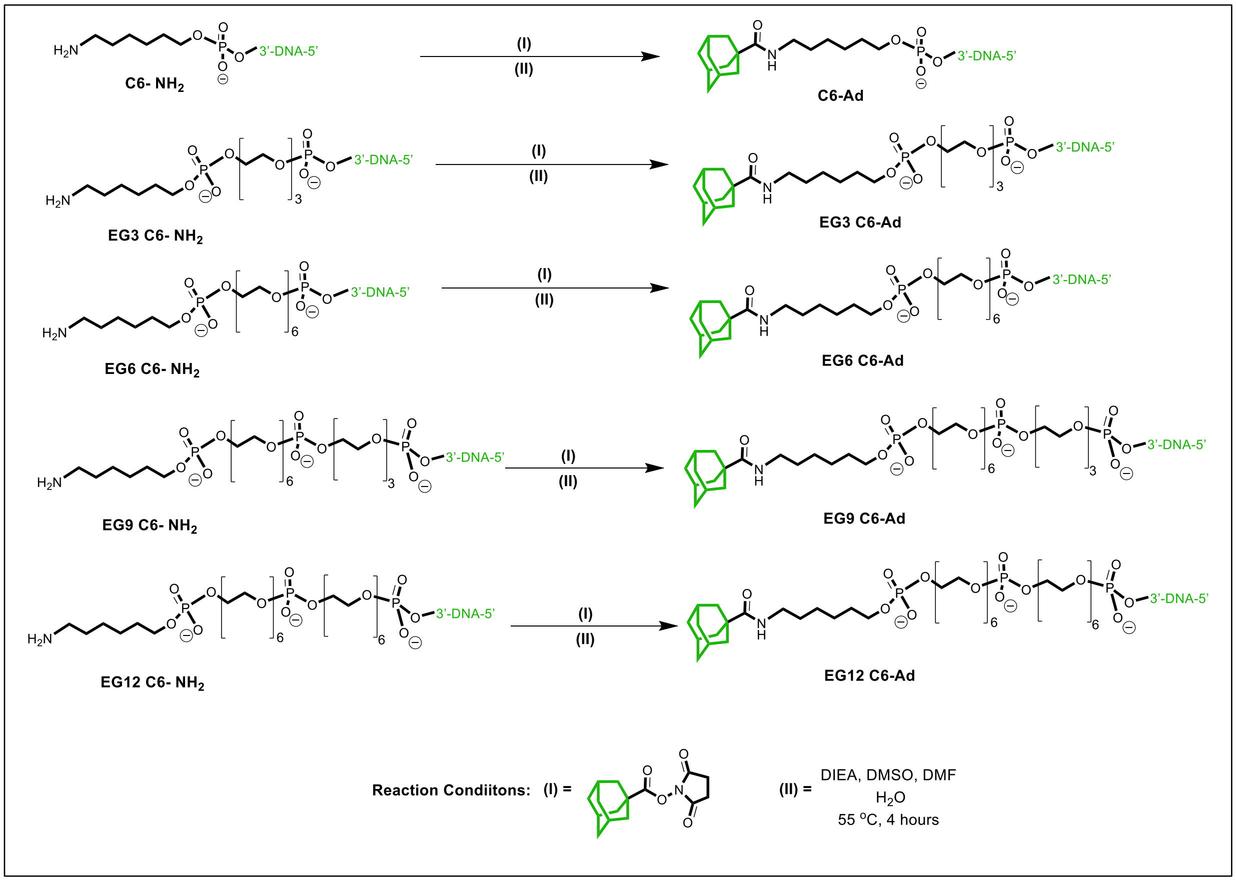

Supplementary Figure 2. Synthesis scheme for Ad-fuel strands; **C6-Ad**, **EG3 C6-Ad**, **EG6 C6-Ad**, **EG9 C6-Ad** and **EG12 C6-Ad**.

A solution of 3 mg (10.8 µmol) adamantane-NHS-ester[1] in 50 µL in (1:2) DMSO: DMF was added to an eppendorf tube containing 0.1 µmol 3' amino modified precursor DNA in 100 µL H_2O and 10 µL DIEA. The solution was incubated at 55 °C for 4 hours and then the solvents were removed using a speedvac concentrator. The resulting crude residue was dissolved in ultrapure water, desalted, purified by RP-HPLC, and analyzed by MALDI-TOF.

This general protocol was followed for the synthesis, purification, and characterization of **C6-Ad**, **EG3 C6-Ad**, **EG6 C6-Ad**, **EG9 C6-Ad**, and **EG12 C6-Ad** input strands. See Supplementary Figure 7-11, 22, and 27 for their characterization details.

S2.3. Synthesis of Phenylalanine-conjugated DNA

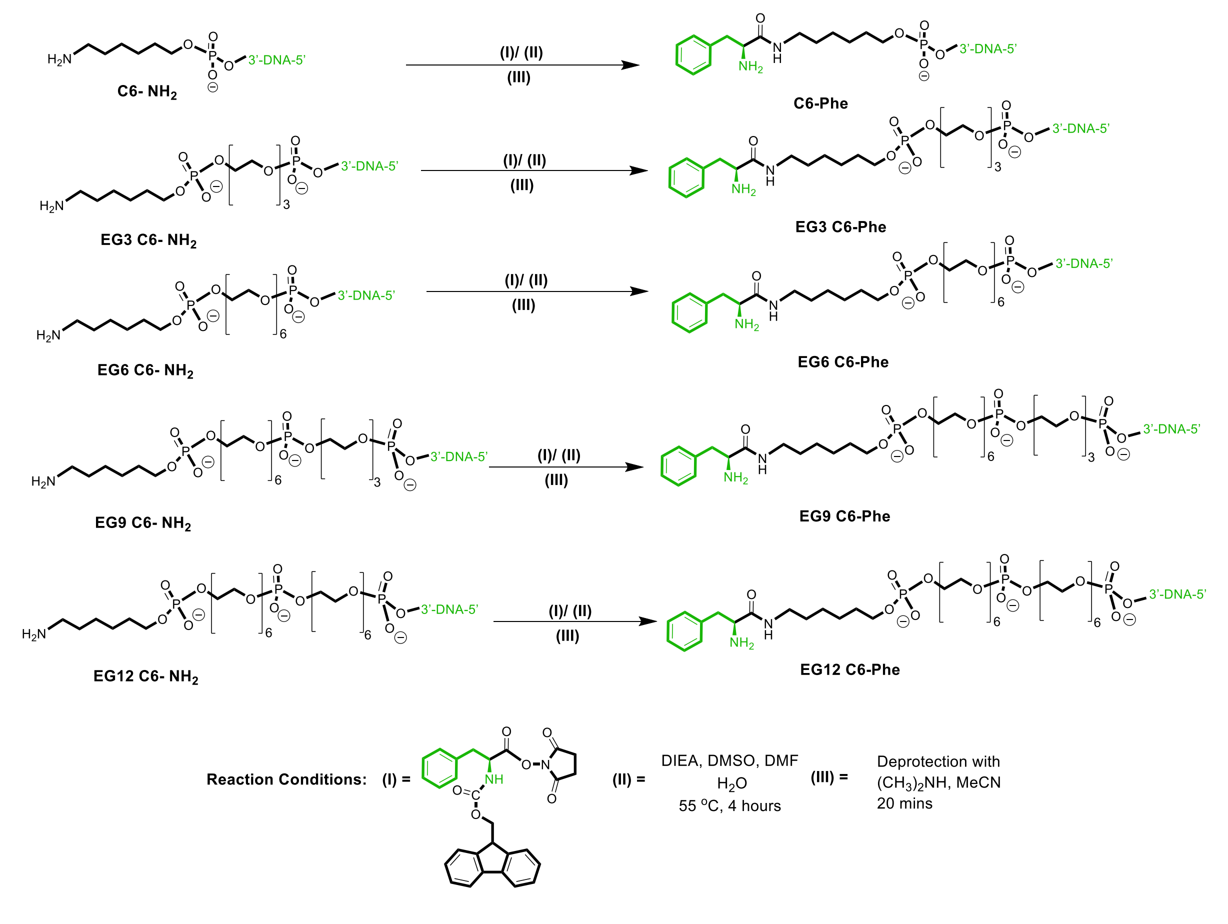

Supplementary Figure 3. Synthesis scheme for Phe-fuel strands; **C6-Phe**, **EG3 C6-Phe**, **EG6 C6-Phe**, **EG9 C6-Phe** and **EG12 C6-Phe**.

A solution of 4 mg (8 µmol) Fmoc-L-Phenylalanine-NHS-ester (Chem Index) in 50 µL of (1:2) DMSO: DMF was added to an eppendorf tube containing 0.1 µmol amino modified precursor DNA in 100 µL H_2O and 10 µL DIEA. The solution was incubated at 55 °C for 4 hours and then the solvents were removed using a speedvac concentrator. The resulting crude residue was dissolved in a 1:1 solution of methylamine and acetonitrile and shaken at RT for 20 min, for the deprotection of the Fmoc group. Then the mixture was co-evaporated with acetonitrile using a speedvac concentrator. The final residue was dissolved in ultrapure water, desalted, purified by RP-HPLC, and analyzed by MALDI-TOF.

This general protocol was followed for the synthesis, purification, and characterization of **C6-Phe**, **EG3 C6-Phe**, **EG6 C6-Phe**, **EG9 C6-Phe**, and **EG12 C6-Phe** fuel strands. See Supplementary Figure 12-16, and 23 for their characterization details.

S2.4. Synthesis of 4-(aminomethyl) benzenesulfonamide and 4-(aminoethyl) benzenesulfonamide conjugated DNA

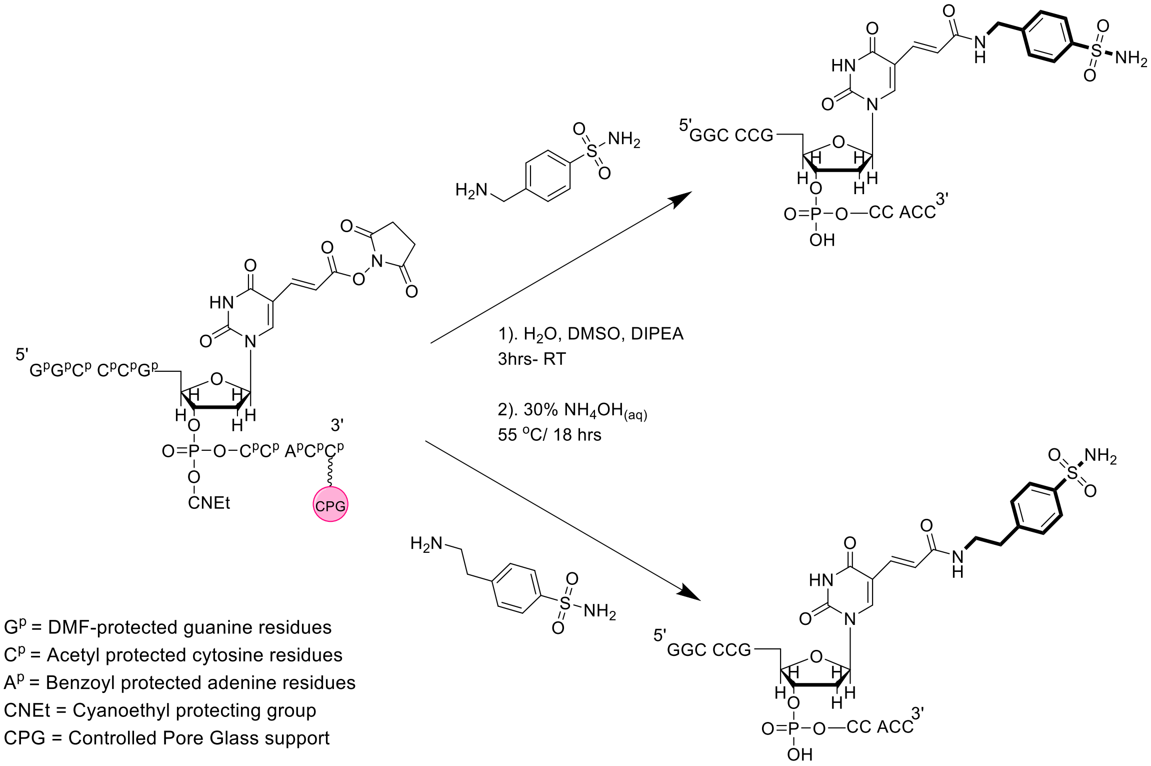

Supplementary Figure 4. Synthesis of **Ei** (n=1); 4-(aminomethyl) benzenesulfonamide conjugated DNA (top) and, **Ei** (n=2); 4-(aminoethyl) benzenesulfonamide conjugated DNA (bottom).

The sulfonamide headgroup containing DNA sequences, **Ei** (n=1 and n=2), were synthesized as follows: Inhibitor small molecule (13.5 µmol) was dissolved in 100 µL 1:1 H_2O/DMSO. This solution was added to 0.3 µmol NHS-dT modified oligonucleotide (on CPG beads) followed by the addition of 10 µL DIPEA. The mixture was stirred at room temperature for 3 hours. After centrifugation and removal of the supernatant, the CPG beads were washed with 3 × 1000 µL DMSO and 3 × 1000 µL H_2O. The oligonucleotide sequence was then cleaved from the beads and globally deprotected with 1000 µL of ammonium hydroxide (30%) at 55°C for 18 hours. The supernatant was separated and dried with a speedvac concentrator. The resulting residue was dissolved in 100 µL H_2O, desalted with a G-25 spin column, purified via RP-HPLC, and characterized with MALDI-TOF. See Supplementary Figure 19 and 20 for their characterization details.

S2.5. RP-HPLC purification of DNA conjugates

All functionalized DNA sequences were purified via sephadex resin microspin G-25 columns (Sigma Aldrich) followed by chromatographic separation using RP-HPLC. The RP-HPLC purification of the synthetic DNA samples were carried out using a Varian Prostar HPLC system, equipped with an Agilent PLRP-S 100 Å 5 μm 4.6 × 250 mm reverse phase column. The column was maintained at 65 °C for all runs. The flow rate was set at 1 mL/min. A gradient composed of two solvents (solvent A is 0.1 M TEAA in 5% acetonitrile and solvent B is 100% acetonitrile) was used and UV-vis absorption was monitored at 260 nm. It is assumed that the difference in extinction coefficients (at 260 nm) of starting material and product species is negligible. The HPLC traces shown in the Supplementary Information S3 were obtained by re-running the already purified samples. The purity percentages were obtained by using the integrated peak areas of all the peaks in the RP-HPLC trace. The concentrations of purified DNA samples were quantified based on their UV-vis absorption at 260 nm and their molar extinction coefficients obtained by nearest neighbor calculations.

S2.6. MALDI-TOF MS analysis of DNA conjugates

All RP-HPLC purified fractions were concentrated with a speedvac concentrator and redissolved in ultrapure water prior to MALDI analysis. MALDI-TOF MS characterization was acquired using a Bruker Autoflex III matrix-assisted laser desorption time-of-flight mass spectrometer (MALDI-TOF MS) or a Bruker Daltonics Autoflex Speed MALDI TOF/TOF, with positive ion and linear detection modes. For all synthetically functionalized DNA samples, a solution of 3-hydroxypicolinic acid (50 mg/mL) and ammonium citrate (10 mg/mL) in 1:1 water/acetonitrile with 0.1% trifluoroacetic acid was used as the matrix. These MALDI samples were prepared by combining 1 μL of DNA solution (~1 mM in H_2O) and 2 μL of matrix solution.

S3. Characterization of Small Molecule-DNA Conjugates

S3.1 Characterization of DNA conjugates used in the development of HG-TMSD

Characterization of R; modified with 6-fluorescein serinol phosphoramidite and 3' dabcyl CPG

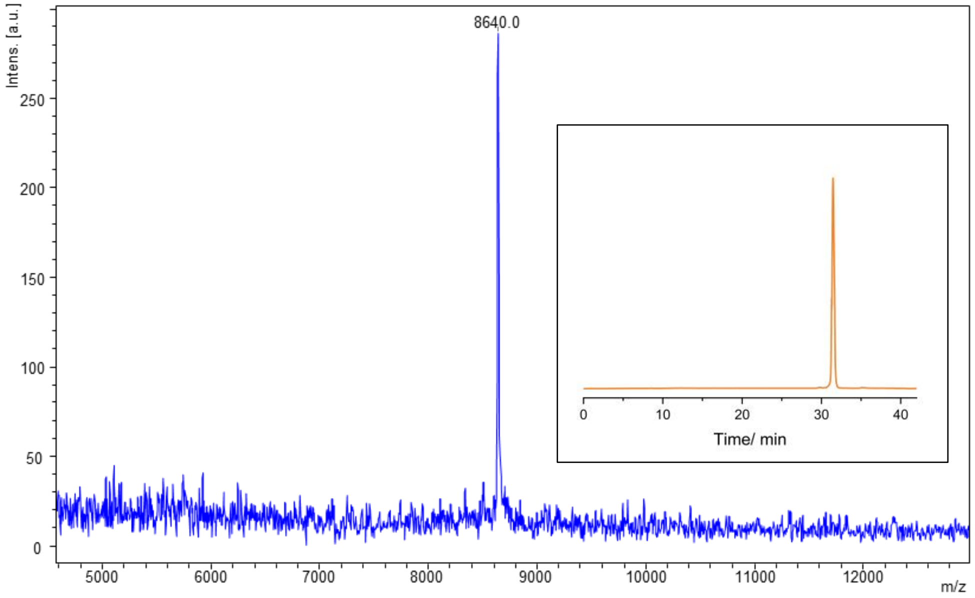

Supplementary Figure 5. MALDI-TOF Spectrum of **R** (blue); observed MW = 8640.0 Da, calculated MW = 8646.9 Da for $[M+ K]^+$. **Inset:** RP-HPLC trace of **R** (orange); purity obtained = 99%.

Characterization of S

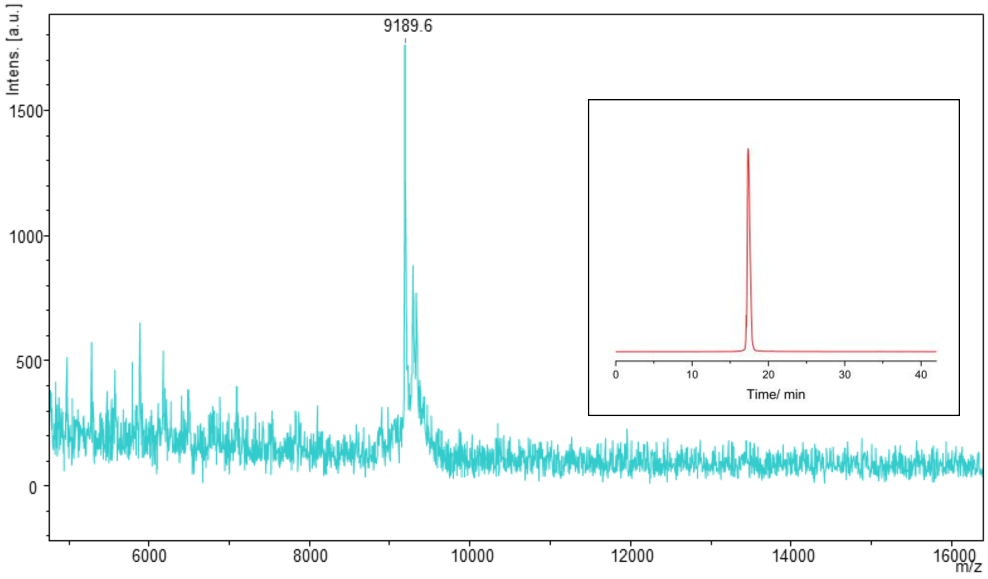

Supplementary Figure 6. MALDI-TOF Spectrum of **S** (blue); observed MW = 9189.6 Da, calculated MW = 9187.3 Da for $[M+ Na]^+$. **Inset:** RP-HPLC trace of **S** (red); purity obtained = 98%.

Characterization of C6-Ad

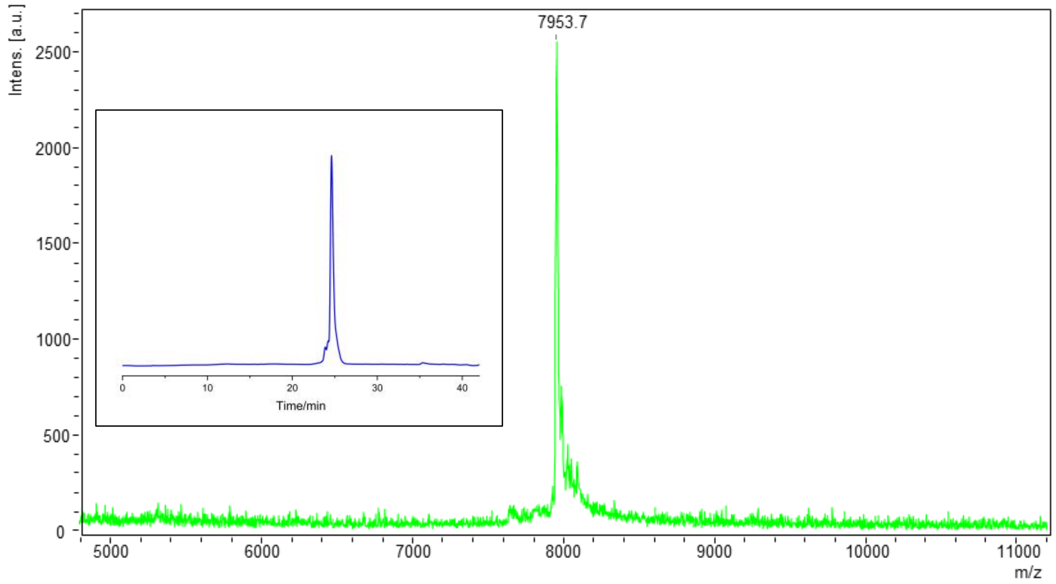

Supplementary Figure 7. MALDI-TOF Spectrum of **C6-Ad** (green); observed MW = 7953.7 Da, calculated MW = 7956.4 Da for $[M+ Na]^{+}$. **Inse**t: RP-HPLC trace of **C6-Ad** (blue); purity obtained = 97%.

Characterization of EG3 C6-Ad

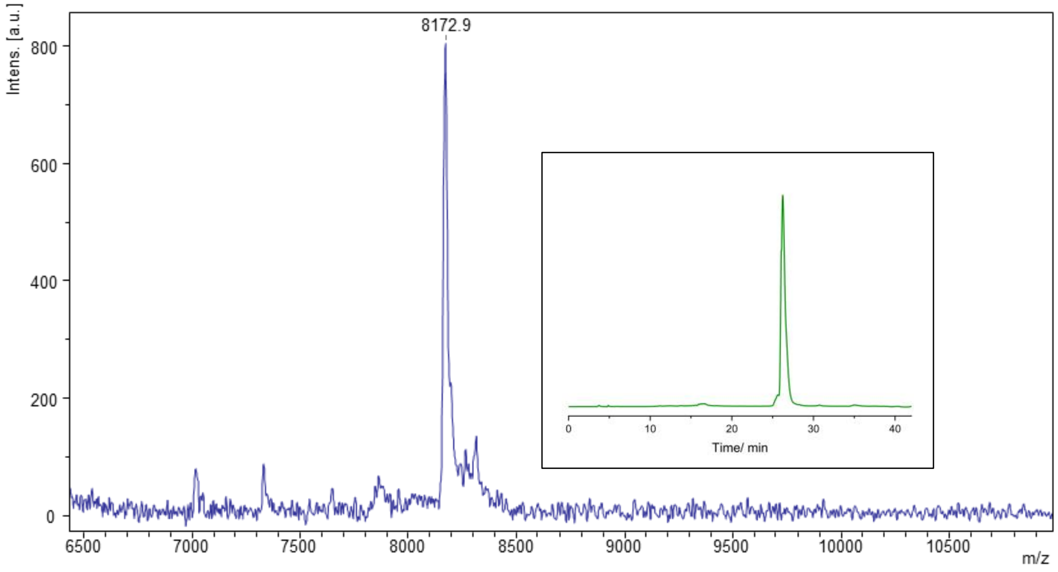

Supplementary Figure 8. MALDI-TOF Spectrum of **EG3 C6-Ad** (blue); observed MW = 8172.9 Da, calculated MW = 8168.5 Da for $[M+ Na]^{+}$. **Inset:** RP-HPLC trace of **EG3 C6-Ad** (green); purity obtained = 95%.

Characterization of EG6 C6-Ad

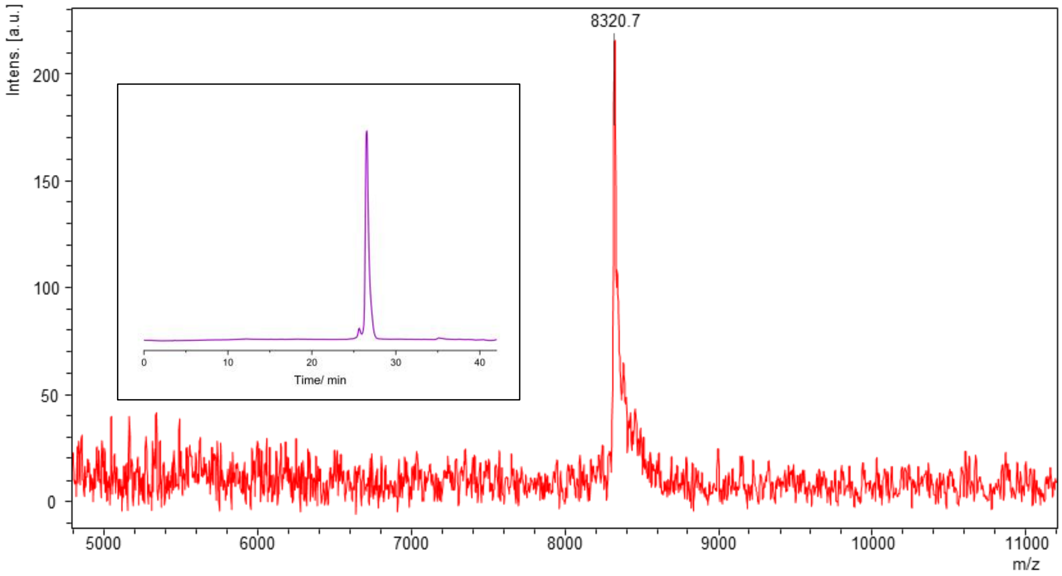

Supplementary Figure 9. MALDI-TOF Spectrum of **EG6 C6-Ad** (red); observed MW = 8320.7 Da, calculated MW = 8316.7 Da for $[M+ K]^{+}$. **Inset:** RP-HPLC trace of **EG C6-Ad** (violet); purity obtained = 95%.

Characterization of EG9 C6-Ad

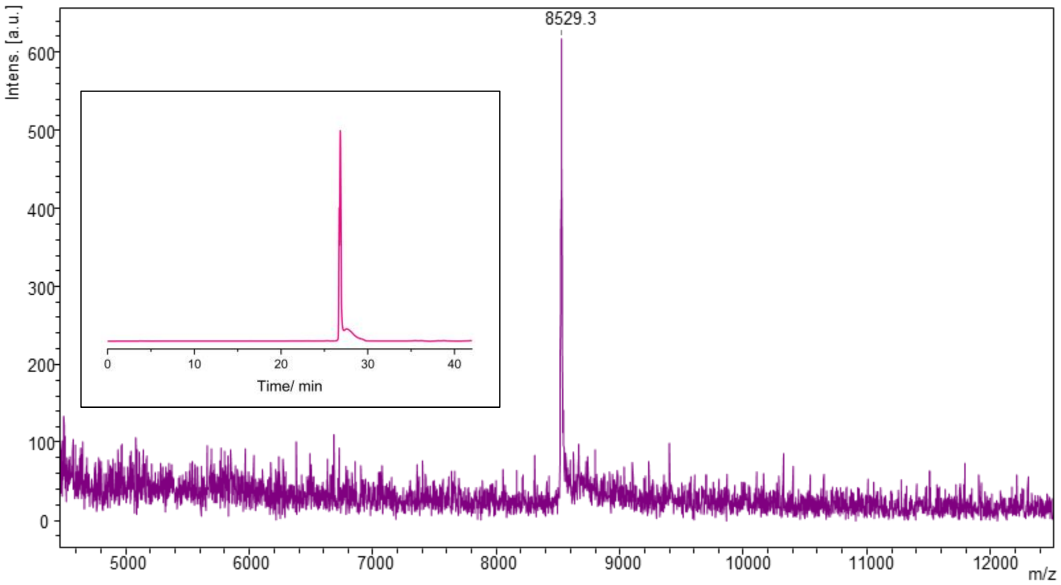

Supplementary Figure 10. MALDI-TOF Spectrum of **EG9 C6-Ad** (purple); observed MW = 8529.3 Da, calculated MW = 8528.8 Da for $[M+ K]^{+}$. **Inset:** RP-HPLC trace of **EG9 C6-Ad** (pink); purity obtained = 87%.

Characterization of EG12 C6-Ad

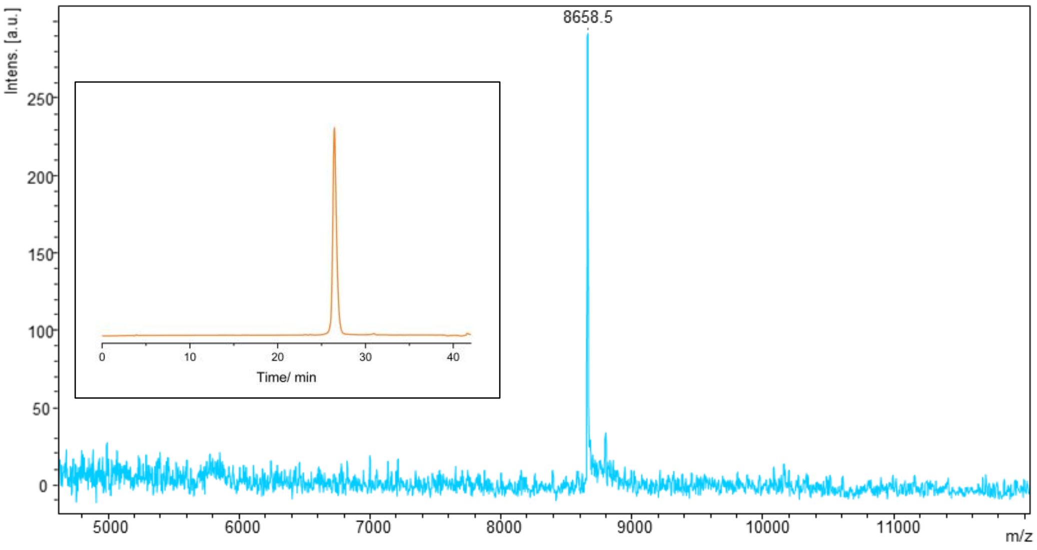

Supplementary Figure 11. MALDI-TOF Spectrum of **EG12 C6-Ad** (blue); observed MW = 8658.5 Da, calculated MW = 8660.9 Da for $[M+K]^+$. **Inset:** RP-HPLC trace of **EG12 C6-Ad** (orange); purity obtained = 99%.

Characterization of C6-Phe

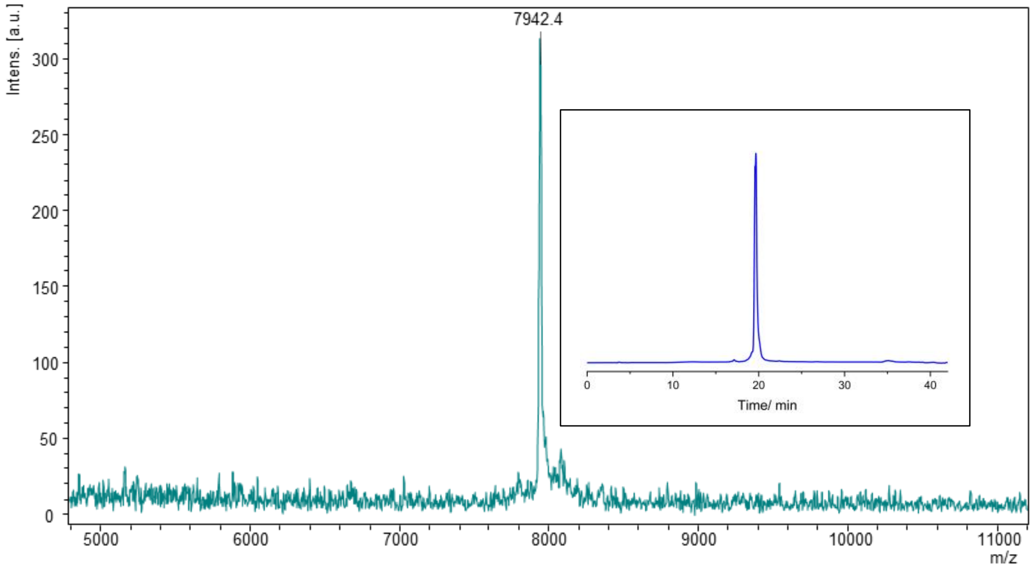

Supplementary Figure 12. MALDI-TOF Spectrum of **C6-Phe** (green); observed MW = 7942.4 Da, calculated MW = 7941.3 Da for $[M+Na]^+$. **Inset:** RP-HPLC trace of **C6-Phe** (blue); purity obtained = 91%.

Characterization of EG3 C6-Phe

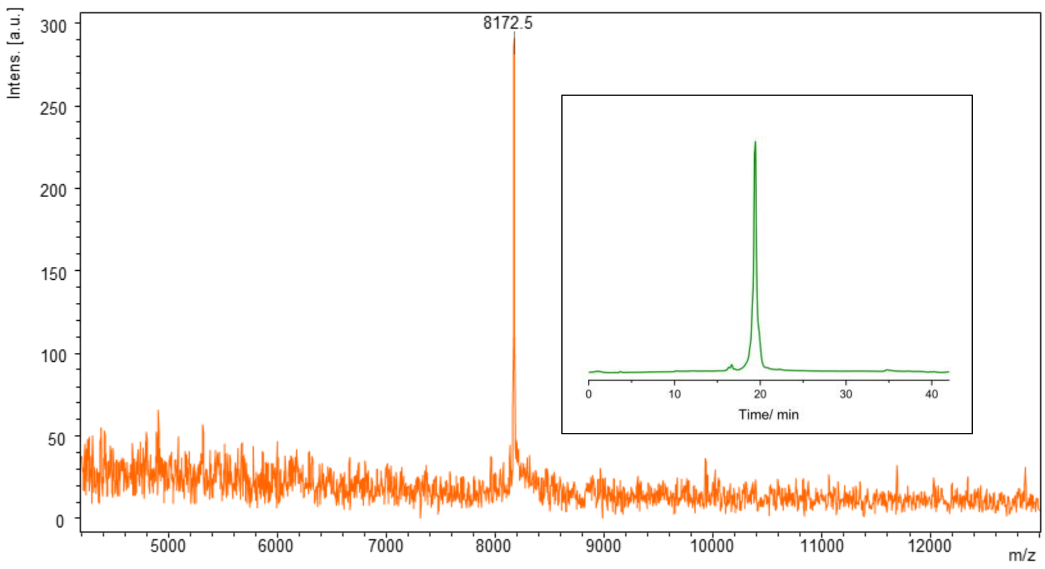

Supplementary Figure 13. MALDI-TOF Spectrum of **EG3 C6-Phe** (orange); observed MW = 8172.5 Da, calculated MW = 8173.5 Da for [M+ Na]$^+$. **Inset:** RP-HPLC trace of **EG3 C6-Phe** (green); purity obtained = 96%.

Characterization of EG6 C6-Phe

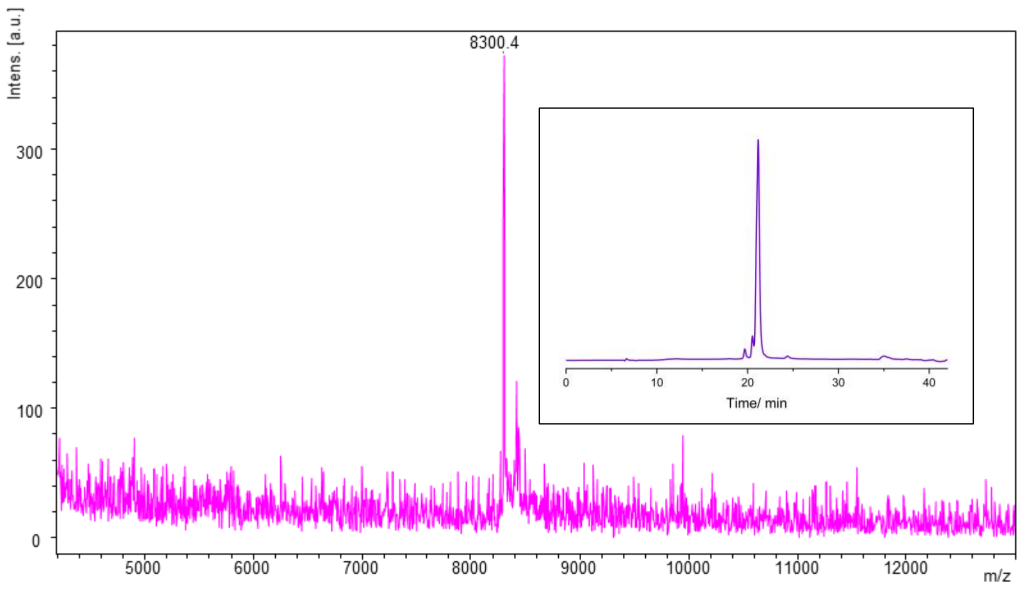

Supplementary Figure 14. MALDI-TOF Spectrum of **EG6 C6-Phe** (pink); observed MW = 8300.4 Da, calculated MW = 8301.6 Da for [M+ K]$^+$. **Inset:** RP-HPLC trace of **EG6 C6-Phe** (purple); purity obtained = 88%.

Characterization of EG9 C6-Phe

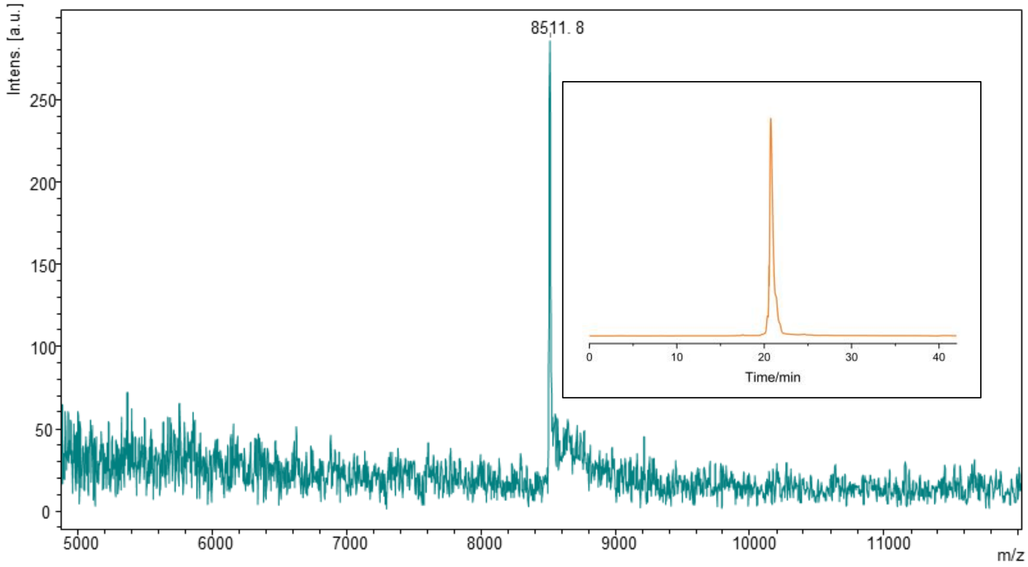

Supplementary Figure 15. MALDI-TOF Spectrum of **EG9 C6-Phe** (green); observed MW = 8511.8 Da, calculated MW = 8513.7 Da for $[M+ K]^+$. **Inset:** RP-HPLC trace of **EG9 C6-Phe** (orange); purity obtained = 96%.

Characterization of EG12 C6-Phe

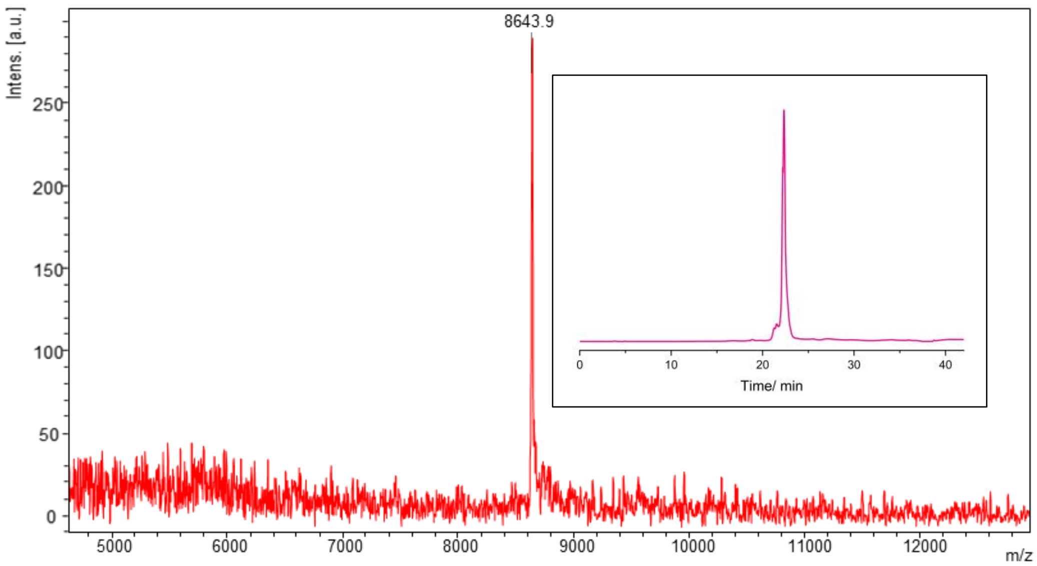

Supplementary Figure 16. MALDI-TOF Spectrum of **EG12 C6-Phe** (red); observed MW = 8643.9 Da, calculated MW = 8645.9 Da for $[M+ K]^+$. **Inset:** RP-HPLC trace of **EG12 C6-Phe** (pink); purity obtained = 91%.

Characterization of S’

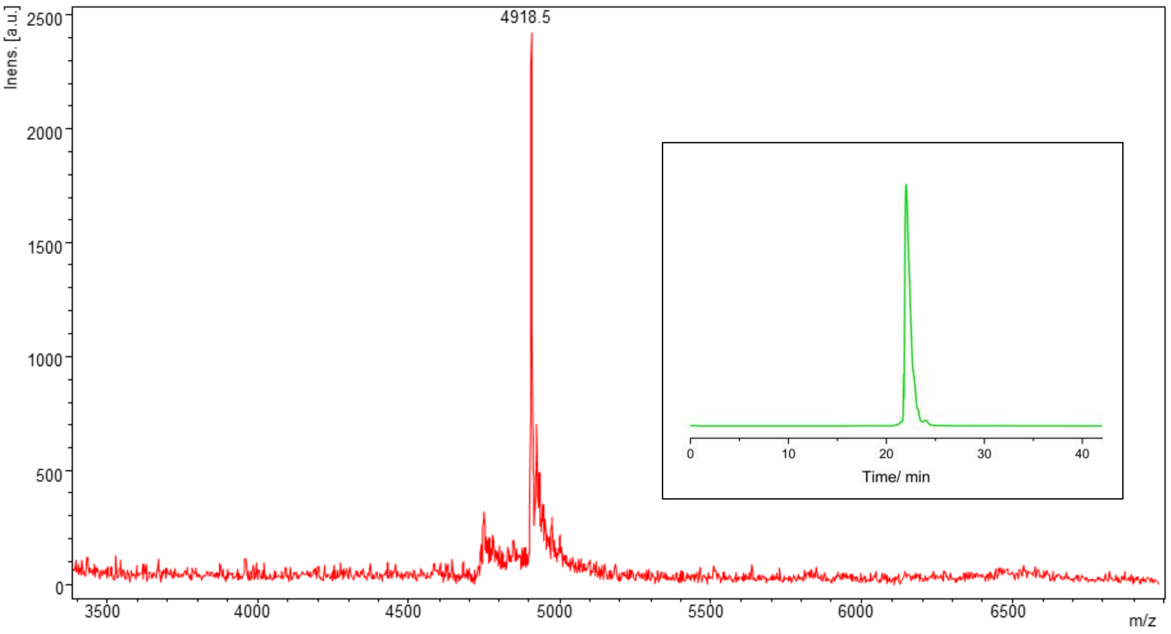

Supplementary Figure 17. MALDI-TOF Spectrum of **S’** strand (red); observed MW = 4918.5 Da, calculated MW = 4917.3 Da for $[M+2H]^{2+}$. **Inset:** RP-HPLC trace of **S’** (green); purity obtained = 95%.

Characterization of I’

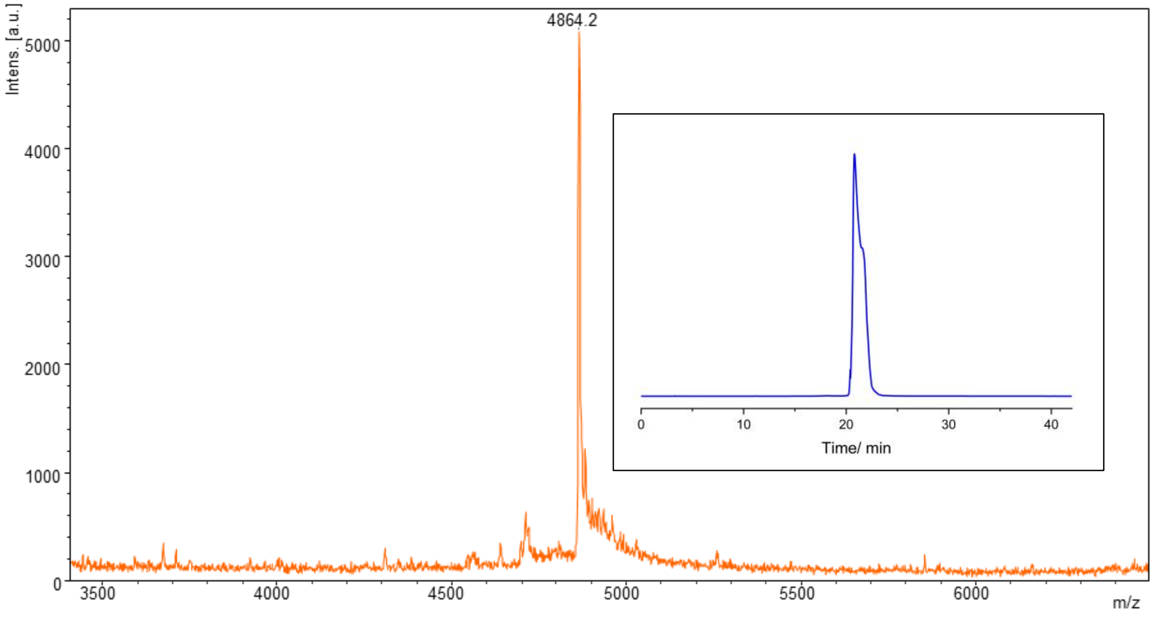

Supplementary Figure 18. MALDI-TOF Spectrum of **I’** strand (orange); observed MW = 4864.2 Da, calculated MW = 4863.8 Da for $[M+2H]^{2+}$. **Inset:** RP-HPLC trace of **I’** (blue); purity obtained = 99%.

S3.2. Characterization of DNA conjugates used in DNA nanomachines that utilize HG-TMSD to control hCA-II activity

Characterization of Ei (n=1)

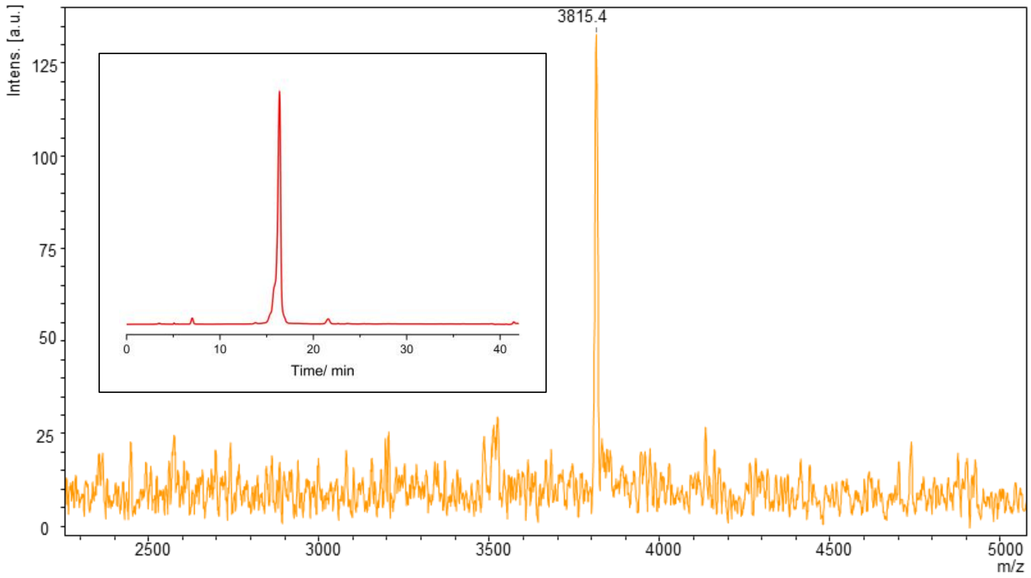

Supplementary Figure 19. MALDI-TOF Spectrum of 4-(aminomethyl) benzenesulfonamide tethered DNA; **Ei (n=1)** (orange), observed MW = 3815.4 Da, calculated MW = 3814.6 Da for $[M+ Na]^+$. **Inset:** RP-HPLC trace of **Ei (n=1)** (red); purity obtained = 96%.

Characterization of Ei (n=2)

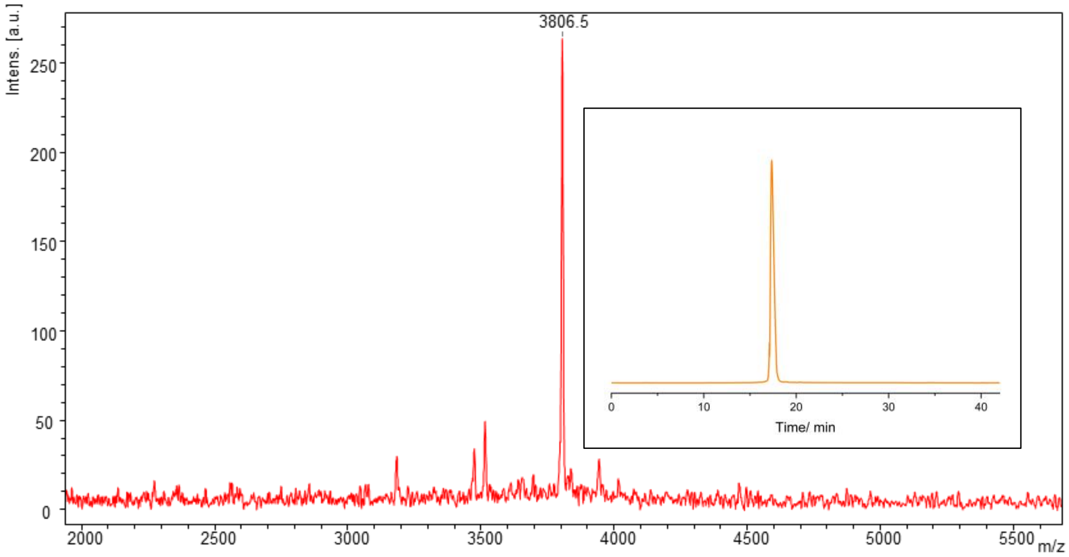

Supplementary Figure 20. MALDI-TOF Spectrum of 4-(aminoethyl) benzenesulfonamide **Ei (n=2)** (red), observed MW = 3806.5 Da, calculated MW = 3806.6 Da for $[M+ H]^+$. **Inset:** RP-HPLC trace of **Ei (n=2)** (orange); purity obtained = 98%.

Characterization of S

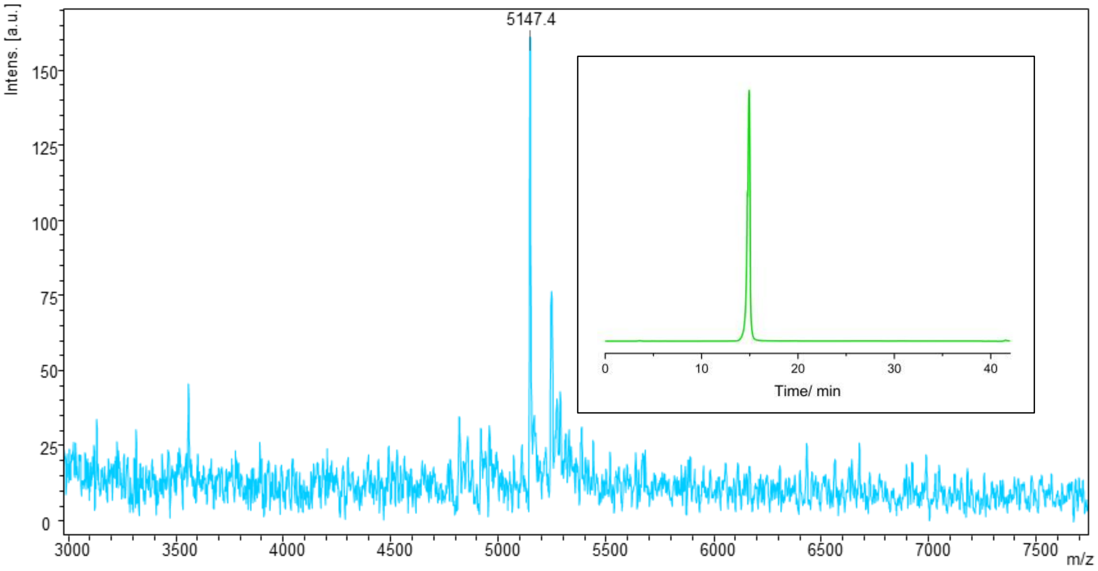

Supplementary Figure 21. MALDI-TOF Spectrum of **S** (blue), observed MW = 5147.4 Da, calculated MW = 5148.7 Da for $[M+H]^+$. **Inset:** RP-HPLC trace of **S** (green); purity obtained = 99%.

Characterization of C6-Ad

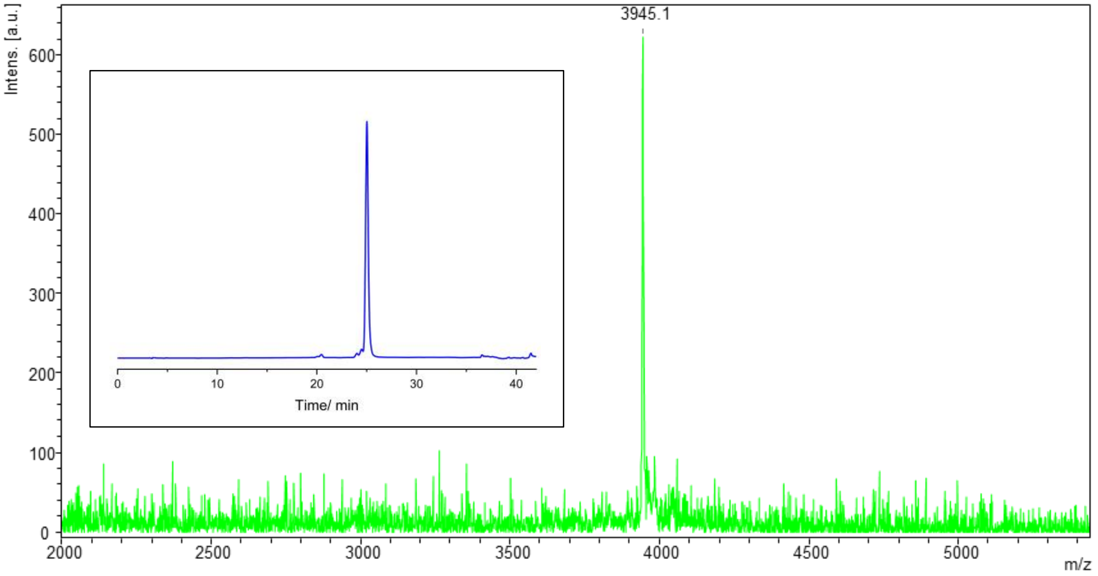

Supplementary Figure 22. MALDI-TOF Spectrum of **C6-Ad** (green), observed MW = 3945.1 Da, calculated MW = 3947.7 Da for $[M+K]^+$. **Inset:** RP-HPLC trace of **C6-Ad** (blue); purity obtained = 89%.

Characterization of C6-Phe

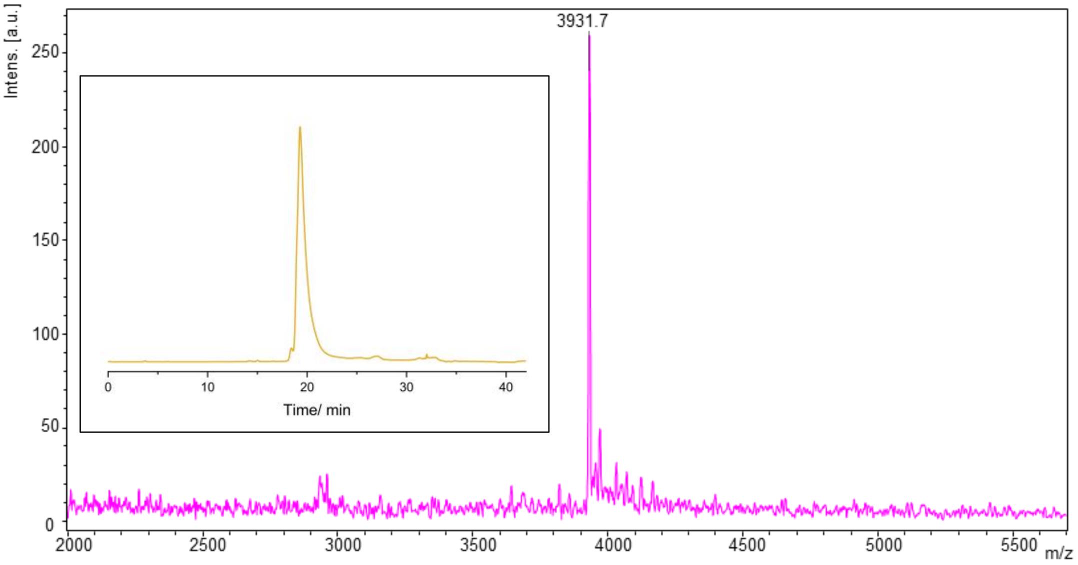

Supplementary Figure 23. MALDI-TOF Spectrum of **C6-Phe** (pink), observed MW = 3931.7 Da, calculated MW = 3932.7 Da for $[M+K]^+$. **Inset:** RP-HPLC trace of **C6-Phe** (yellow); purity obtained = 96%.

Characterization of E*

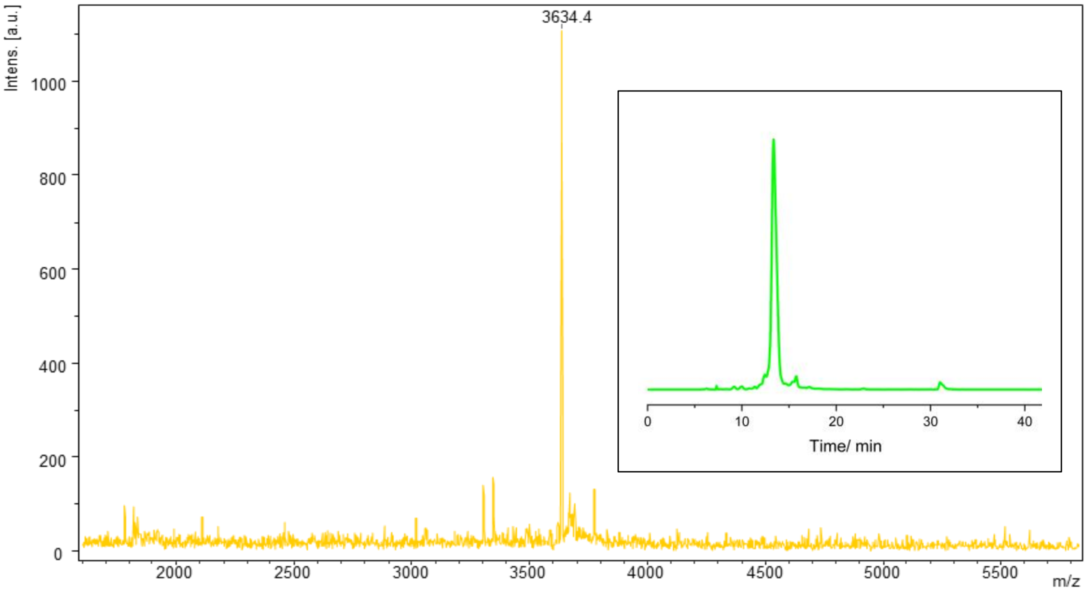

Supplementary Figure 24. MALDI-TOF Spectrum of **E*** (yellow), observed MW = 3634.4 Da, calculated MW = 3640.4 Da for $[M+NH_4]^+$. **Inset:** RP-HPLC trace of **E*** (green); purity obtained = 88%.

3.3. Characterization of DNA conjugates used in sequential BP-TMSD and HG-TMSD reactions to detect microRNA-182

Characterization of R; modified with 6-fluorescein serinol phosphoramidite and 3' dabcyl CPG

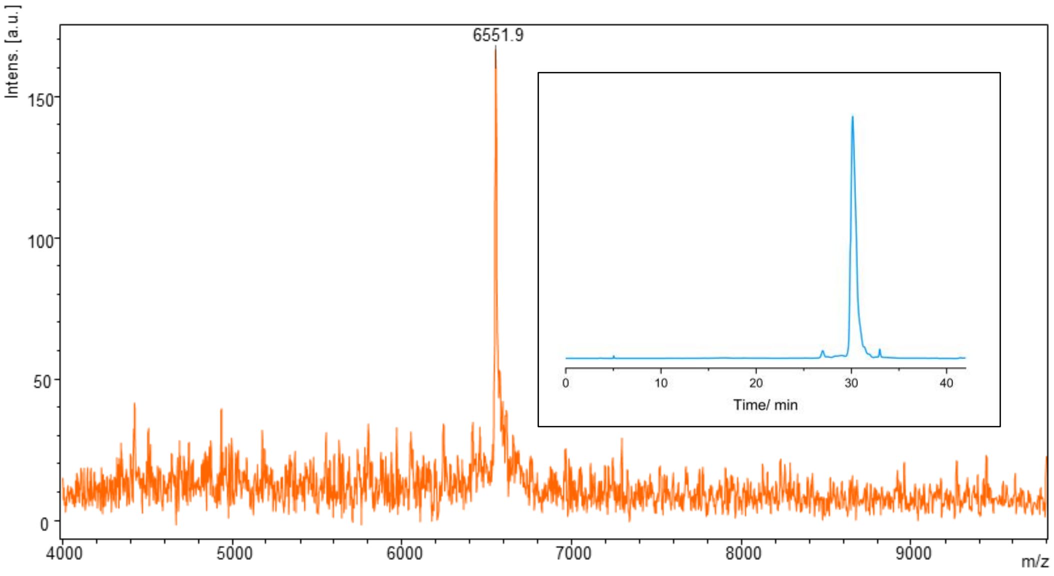

Supplementary Figure 25. MALDI-TOF Spectrum of **R** (orange), observed MW = 6551.9 Da, calculated MW = 6545.5 Da for $[M+ K]^+$. **Inset:** RP-HPLC trace of **R** (blue); purity obtained = 96%.

Characterization of S

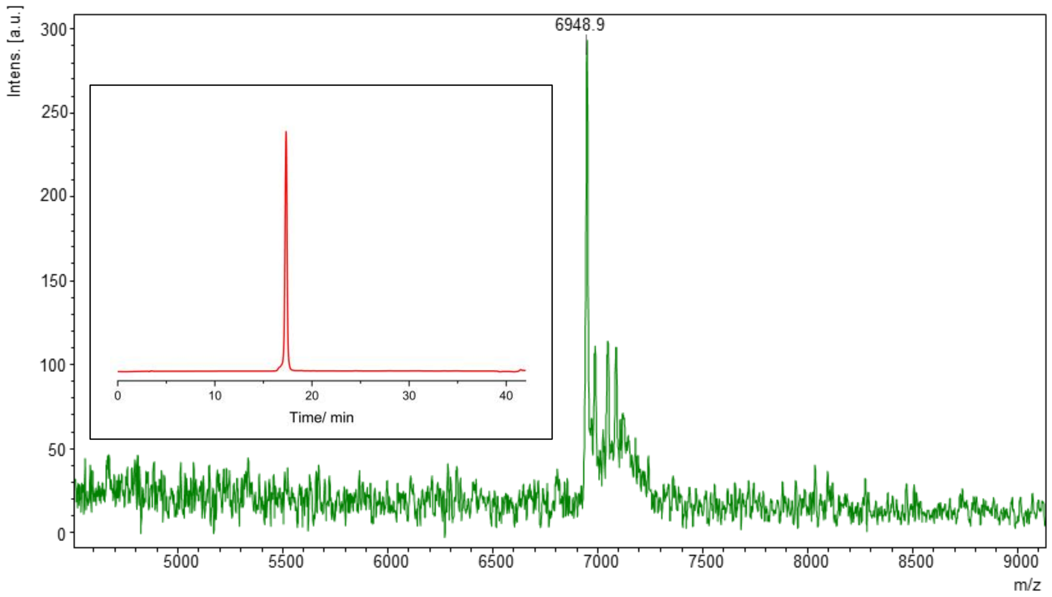

Supplementary Figure 26. MALDI-TOF Spectrum of **S** (green), observed MW = 6948.9 Da, calculated MW = 6947.8 Da for $[M+ Na]^+$. **Inset:** RP-HPLC trace of **S** (red); purity obtained = 99%.

Characterization of C6-Ad

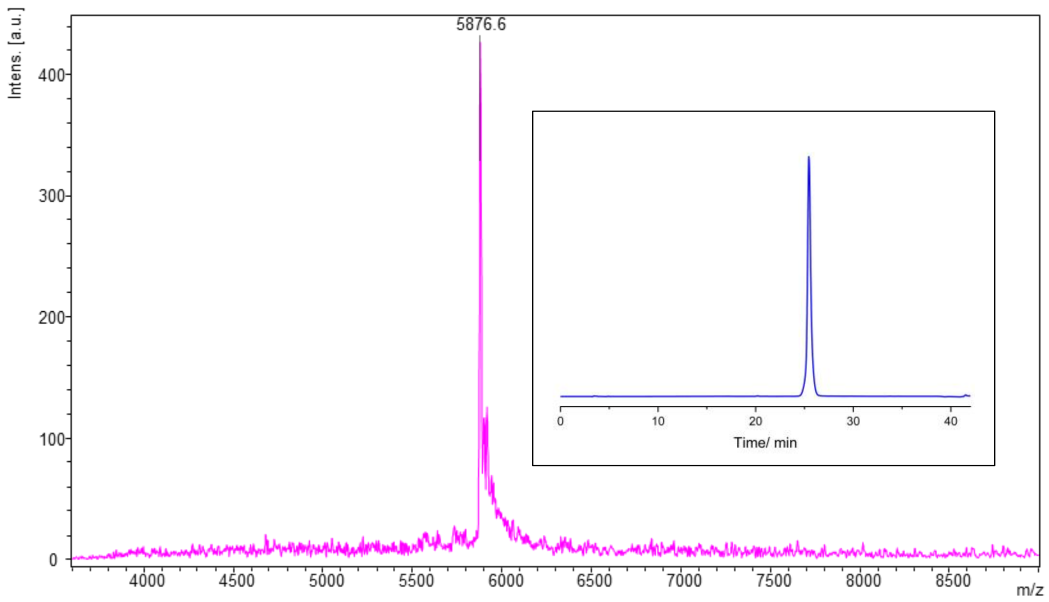

Supplementary Figure 27. MALDI-TOF Spectrum of **C6-Ad** (pink), observed MW = 5876.6 Da, calculated MW = 5871.0 Da for $[M+K]^+$. **Inset:** RP-HPLC trace of **C6-Ad** (blue); purity obtained =99%.

Characterization *of A*

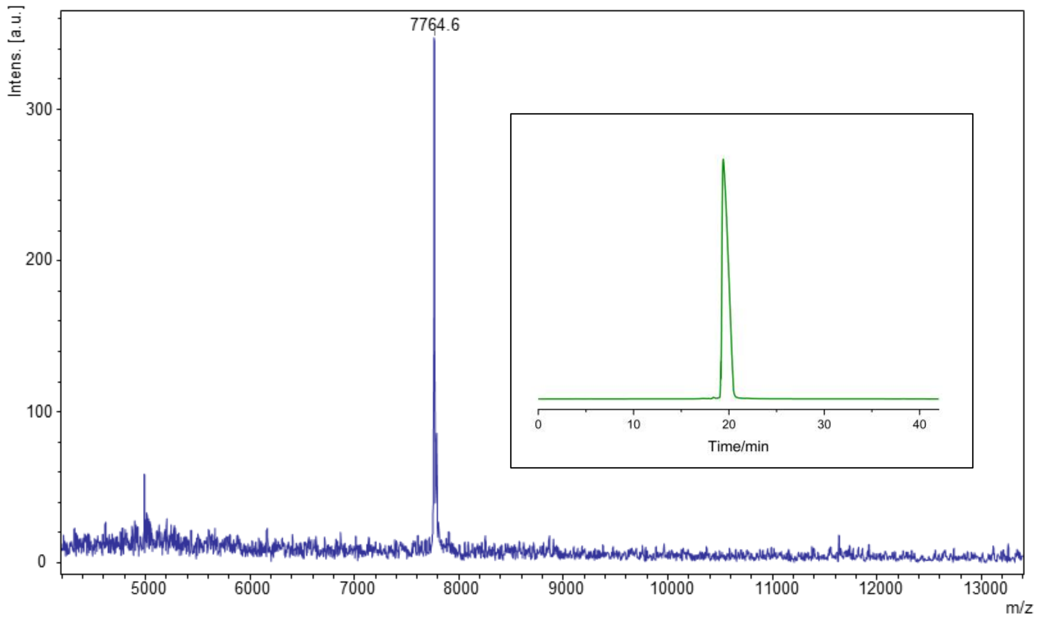

Supplementary Figure 28. MALDI-TOF Spectrum of **A** (blue), observed MW = 7764.6 Da, calculated MW = 7757.3 Da for $[M+H]^+$. **Inset:** RP-HPLC trace of **A** (green); purity obtained = 99%.

Characterization of A'; modified with 5' biotin phosphoramidite and 3' fluorescein CPG

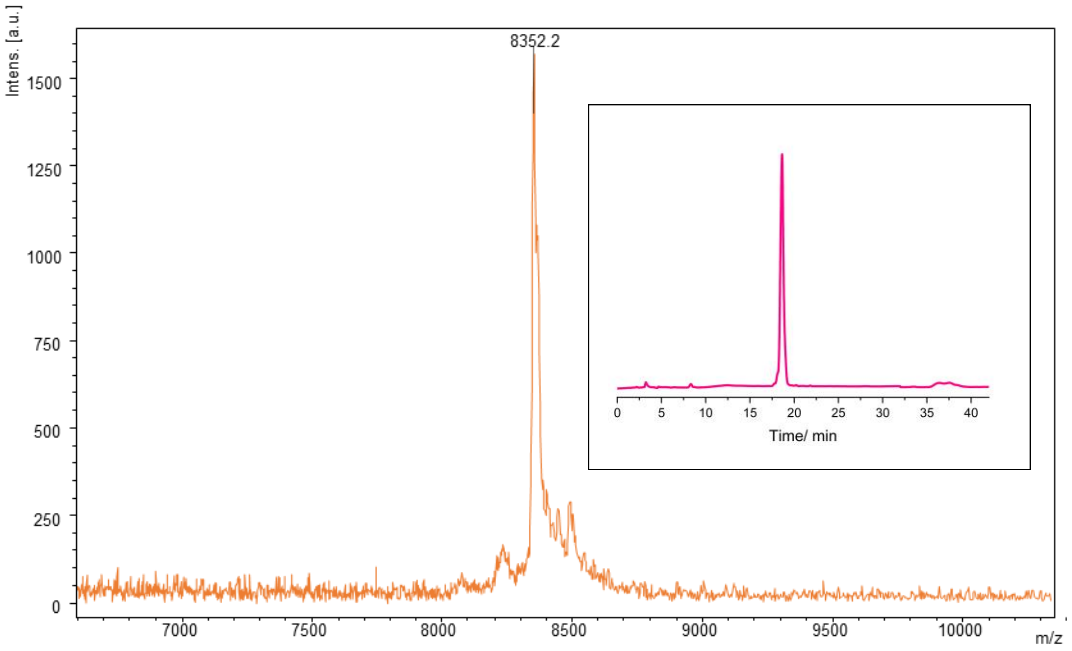

Supplementary Figure 29. MALDI-TOF Spectrum of **A'** (orange), observed MW = 8352.2 Da, calculated MW = 8349.7 Da for $[M+ K]^+$. **Inset:** RP-HPLC trace of **A'** (pink); purity obtained =92%.

S4. Supporting Figures

S4.1. Comparison of HG-TMSD displacement kinetics with conventional BP-TMSD

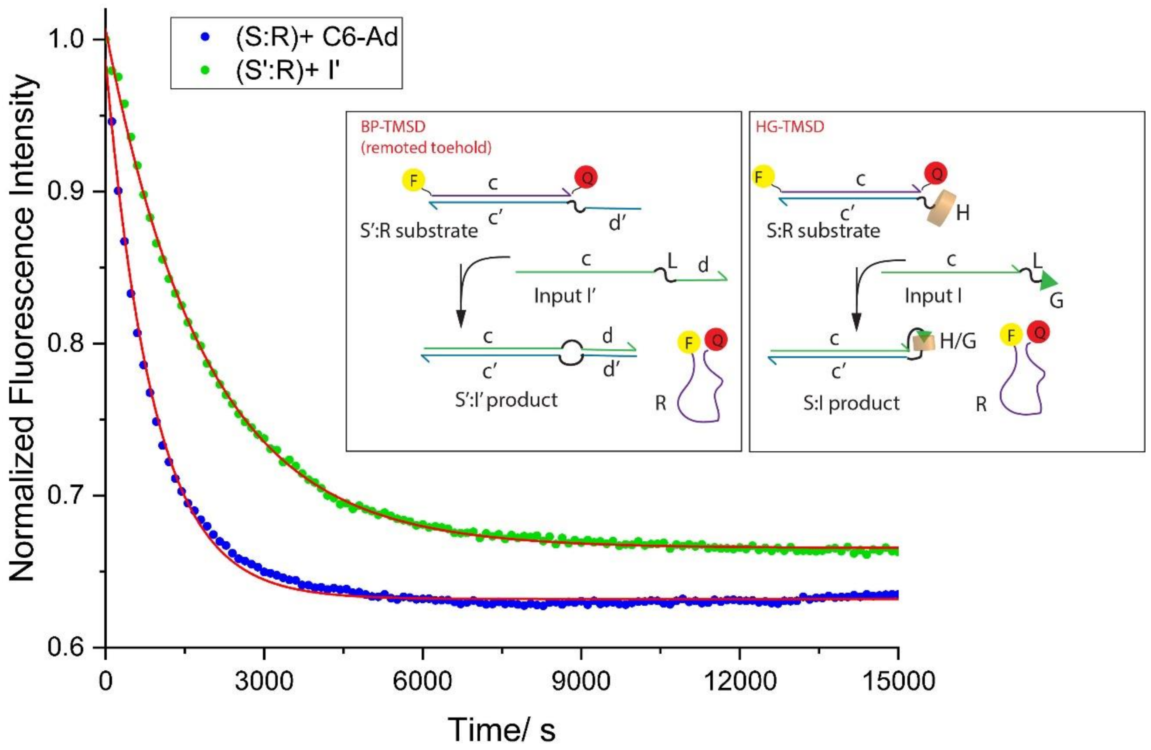

Supplementary Figure 30. Comparison of HG-TMSD with analogous BP-TMSD containing a remote toehold. Fitted kinetic decay profiles for HG-TMSD; **S:R** +10 eq. of **C6-Ad** (i.e., Input I), (blue profile), and the BP-TMSD analog with a remote toehold (6 base toehold) containing a 12 carbon linker (L); **S':R** + 10 eq. of **I'** (green profile). The data were fit to equation 1 (fitted lines are represented in red). **S'**= substrate strand for BP-TMSD with 6 base toehold sequence d' that is separated from the 25 nucleotide core DNA domain c' by a 12-carbon linker. **I'** = input strand for BP-TMSD with complementary 6 base toe sequence d that is separated from the 25 nucleotide core DNA domain c by a 12-carbon linker (L). The buffer used in the displacement studies was: 20 mM Tris, 10 mM NaCl, 5 mM $MgCl_2$, pH = 7.5. Results are the mean of 3 independent experiments. **Inset:** General scheme for HG-TMSD and BP-TMSD with remote toehold.

In an effort to gauge how HG-TMSD compares to BP-TMSD, we prepared an analogous BP-TMSD system (**S':R** + **I'**), shown in the inset. Here, the substrate strand **S'** contains the core DNA domain (c') but incorporates a 6-base toehold (d'). Since in the HG-TMSD system, the substrate **S** includes a synthetic linker (containing 12 bonds) between the CB7 toehold and the core DNA domain, we also included a C12

linker separating the toehold and the core DNA domains in **S'**. Similarly, the input sequence **I'** also contains a C12 linker separating the core domain c and the 6-base toe d.

Since 10 eq. of input sequence was used (compared to substrate duplex), we fit the kinetic data to a pseudo-first order equation (1) and the apparent rate constant *k* was determined. Here *F* = fluorescence at time *t*, *F0* = initial fluorescence and *F1*= final fluorescence.[5]

$$\boldsymbol{F = F1 + (F0 - F1)\, e^{-kt}} \qquad \textbf{(Eq. 1)}$$

Interestingly, when 10 eq of **I'** was used, the BP-TMSD reaction exhibited a $t_{0.8}$ of 1.72 ± 0.07 x 10^3 s and a corresponding pseudo first order rate constant of 5.29 ± 0.20 x 10^{-4} s^{-1} (which is 2 times slower than for the HG-TMSD reaction featuring the **C6-Ad** input; *k* = 1.11 ± 0.07 x10^{-3} s^{-1}).

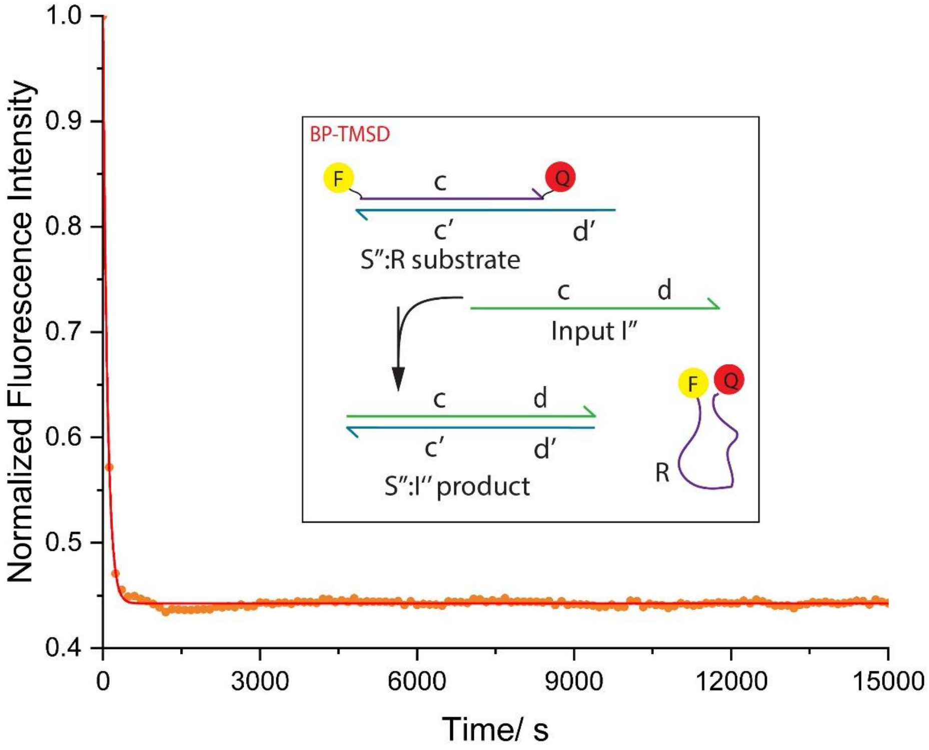

Supplementary Figure 31-i. Real time kinetic decay profile for conventional BP-TMSD without a remote toehold. Shown in the inset is a general scheme for BP-TMSD. Substrate duplex **S'': R** (1 µM) composed of substrate **S''** (with sequence c' + d', where d' is a 6-base toehold) is hybridized with the reporter strand **R** (with sequence c). Upon introduction of 10 eq. of input DNA (**I''**) carrying a complementary 6 base toe sequence d and core sequence c, the reporter strand **R** is displaced. The displacement of **R** was monitored over 1.5x10^4 s. The buffer used in the displacement studies was: 20 mM Tris, 10 mM NaCl, 5 mM $MgCl_2$, pH = 7.5. Results are the mean of 3 independent experiments.

We also compared the displacement kinetics of the HG-TMSD system with conventional Watson-Crick-Franklin base-pair derived strand displacement in the absence of a remote toehold. For this purpose, we used a 6 base toehold and toe domain, on the substrate and input strands, respectively (similar to that of the remote toehold BP-TMSD system described above). Here, the apparent rate constant *k* was found to be 1.23 ± 0.17 x 10^{-2} s^{-1}, a value that is 20 times faster than the rate constant observed for BP-TMSD with the remote toehold. This result is consistent with the literature because introduction of spacer groups in BP-TMSD is known to slow down the rate of displacement reactions when compared to those that function via contiguous DNA domains.[6] Interestingly, we noted that the HG-TMSD reaction exhibits displacement kinetics that is in between the conventional BP-TMSD and the remote BP-TMSD. Taken together, these studies demonstrate that the CB7-adamantane host-guest interaction is an effective mechanism for initiating DNA strand displacement.

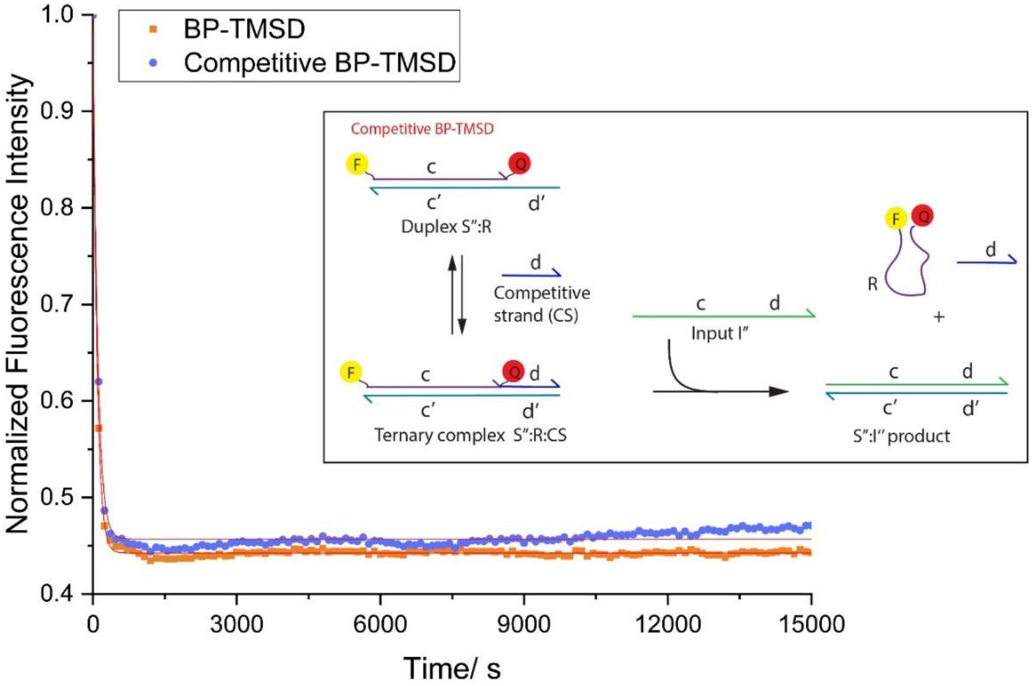

Supplementary Figure 31-ii. Comparison of displacement kinetics decay profiles for BP-TMSD under competitive and non-competitive conditions. For the competitive conditions: 1 eq. of **S":R** duplex (1 µM) was first incubated with 10 eq. of competing **CS** sequence, and upon addition of 10 eq. of **I",** displacement kinetics were monitored over 1.5x10^3 s. The buffer used in the displacement studies was: 20 mM Tris, 10 mM NaCl, 5 mM $MgCl_2$, pH = 7.5. **Inset:** Schematic for competitive BP-TMSD (for a schematic on non-competitive BP-TMSD, see inset, Figure 31-i).

In order to compare the HG-TMSD and BP-TMSD displacement kinetics under the influence of competing agents (see main text Figure 4 for HG-TMSD displacement kinetics in the presence of competing small molecule guests), competitive conditions were mimicked in the conventional BP-TMSD system by addition

of 10 eq. of a 6-nucleotide competitive sequence, CS (with sequence d). Substrate duplex (1 µM) was incubated with 10 eq. of CS for 3 hours, followed by the addition of 10 eq. of input strand (**I''**) containing sequence c + d. The kinetic decay profile in the presence of the CS (blue) was compared to that in the absence of competitive conditions (orange). BP-TMSD under competitive conditions exhibited an apparent rate constant *k* of 1.06 x 10^{-2} s^{-1} which did not significantly differ from the conventional BP-TMSD rate constant in the absence of the competitive conditions (1.23 x10^{-2} s^{-1}). These results suggest that unlike HG-TMSD, the rate of BP-TMSD cannot be readily modulated by using such competitive agents.

S4.2. HG-TMSD mutation studies

In conventional BP-TMSD, the introduction of mutations in the input sequence can lead to decrease in the displacement rate since the base mismatch between the input and substrate strands enhances the rate of the backward reaction of the branch migration step at the mutation site. In this study, **C6-Ad** input sequences with single base mutations were investigated to determine how mismatches influenced the kinetics of HG-TMSD. As shown in Supplementary Figure 32, three different loci were used as mutation sites for the input strands, and these (**M3 C6-Ad**, **M14 C6-Ad**, and **M25 C6-Ad**) are named based on the position of the mismatch base from the 3' end carrying the adamantane guest.

All these sequences were prepared by following the synthetic scheme shown in Supplementary Figure 2, purified with RP-HPLC and characterized with MALDI-TOF. MALDI-TOF observed MW of **M3 C6-Ad** was 7936.0 Da, while the calculated MW was 7931.4 Da for $[M+ Na]^+$, RP-HPLC trace purity obtained was 99%. MALDI-TOF observed MW of **M14 C6-Ad** was 7995.0 Da, while the calculated MW was 7996.4 Da for $[M+ Na]^+$, RP-HPLC trace purity obtained was 94%. MALDI-TOF observed MW of **M25 C6-Ad** was 7999.2 Da, while the calculated MW was 7996.4 Da for $[M+ Na]^+$, RP-HPLC trace purity obtained was 99%.

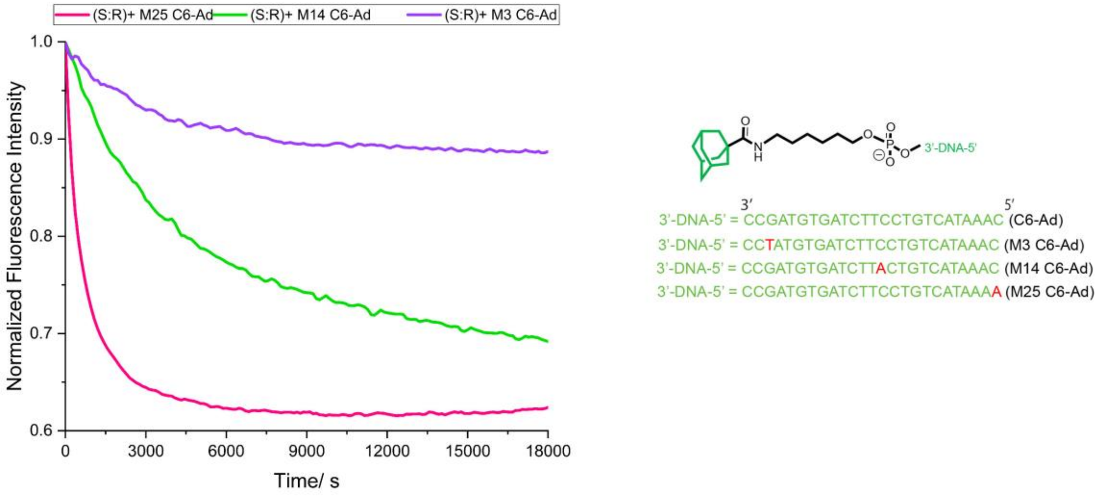

Supplementary Figure 32. Kinetic decay profile following the fluorescence-quenching of the displaced reporter **R** from 1.0 µM duplex **S:R** as a function of 10 equivalents of adamantane-containing mutant input strands. Inset: Location of

the mutation on the input sequence is shown in red. Buffer used in these studies: 20 mM Tris, 10 mM NaCl, 5 mM $MgCl_2$, pH = 7.5.

The fluorescence-quenching experiments show a significant decrease in the reaction rate when the mutation is in proximity to the guest head-group. Specifically, **M3 C6-Ad** (purple line), that has a mutation three residues from the 3’ end, did not reach the 80% signal level. As the mutation is introduced further away from the guest toe, the lesser the effect on the displacement kinetics. For example, the **M14 C6-Ad** (green line), with the mutation near the middle of the input sequence, has a $t_{0.8}$ of 4.4×10^3 s, which is ca. 7 fold slower than the “wild-type” **C6-Ad** ($0.6 \pm 0.1 \times 10^3$ s). Further, when **M25 C6-Ad** (which has the mutation on the 5’ terminus- pink line) is used as the input, no appreciable change in the rate is observed compared to wild-type **C6-Ad**. This latter mutation does not influence the rate of HG-TMSD since the branch migration step is essentially complete (the reporter is already dissociated) when the mismatch base-pairing between **S** and input **M25 C6-Ad** is attempted.

S4.3. Supplementary figures relevant to hCA-II inhibition

S4.3.1.SPR sensograms of Ei (n =2) binding to hCA-II in single stranded and duplex forms

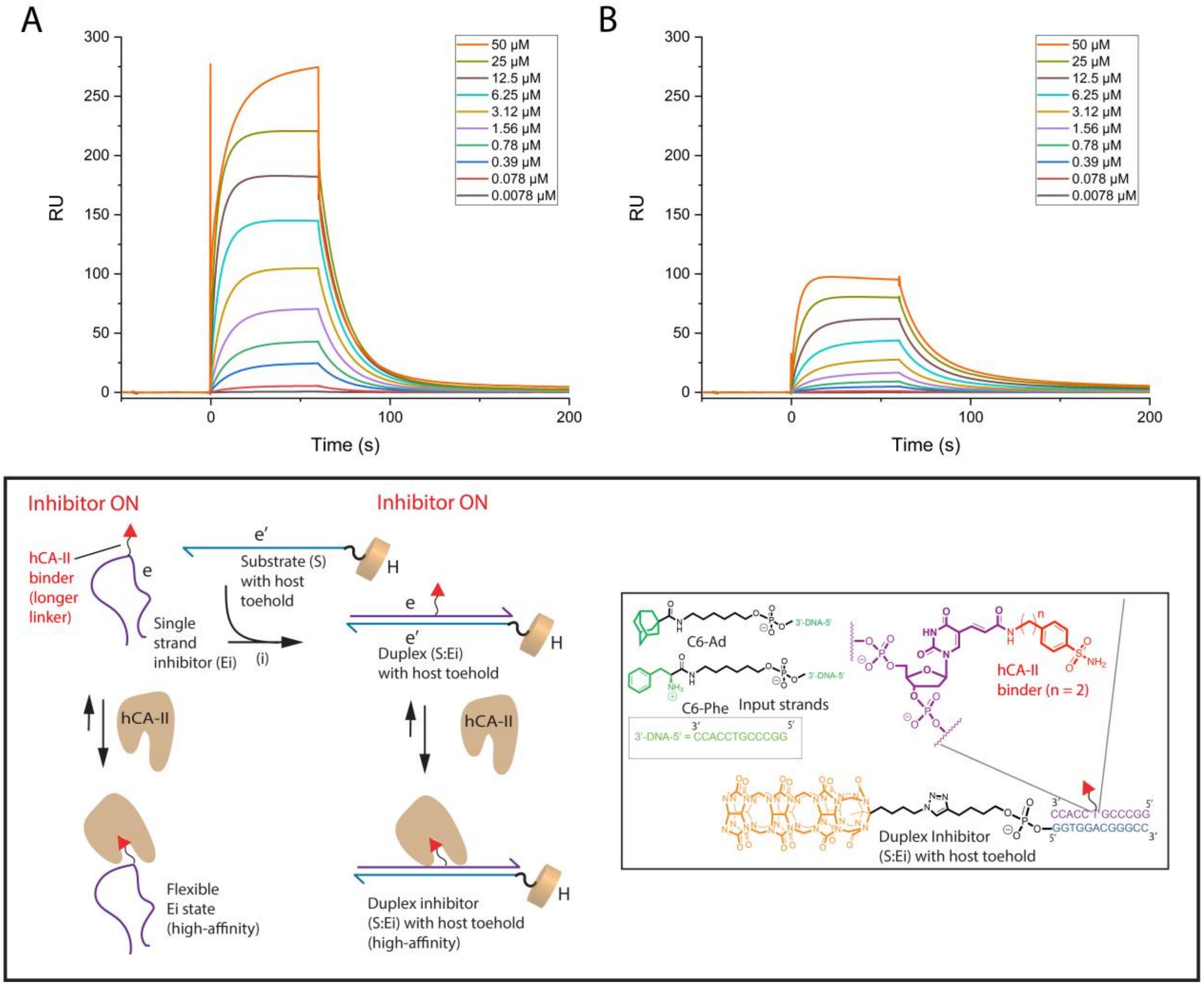

Supplementary Figure 33 (i). (Top) SPR sensograms of (A) single strand Ei (n=2) and (B) duplex S:E$_i$ binding to hCA-II. The concentration of DNA was varied from 7.8x10^{-3} to 50 μM. The running buffer used in the SPR binding assays was PBS-P+ (containing 0.5% surfactant P20, pH 7.4). **(Bottom):** Schematic of the single-strand and duplex states of **Ei** (n=2) binding to hCA-II. **Inset:** Detailed structures of the sequences used.

An important consideration in our design strategy is the length of the linker group bridging the benzenesulfonamide binder and the DNA domain. If the linker is too long, then duplex rigidity may not be sufficient to attenuate the hCA-II binding activity. We thus prepared two versions of **Ei** differing by a methylene group. We performed surface plasmon resonance (SPR) based binding experiments to interrogate the differential binding abilities of single-stranded and duplex states of the two **E$_i$** sequences. The SPR sensograms corresponding to **Ei** with 4-(aminoethyl) benzenesulfonamide (n=2) modification is shown above. The binding constant K_d of **Ei** (n=2) in its single stranded and duplex forms were not significantly different from each other; single stranded version exhibited a K_d of 4.73 x10^{-6} M whereas the

K_d for the **S:Ei** duplex was found to be 7.62 x10^{-6} M. Interestingly, the **Ei** sequence that only has one methylene spacer (n=1) exhibited a K_d of 12.04 x10^{-6} M for the single strand state which is ca. ~3 times weaker than for the corresponding single stranded **Ei** sequence with two methylene spacers.

The Chi^2 values for the kinetic experiments were within a range of 0.14 to 22.70, whereas the U-values were within a range of 1 to 5. Chi^2 values serve as quantitative indication that the data fits well to the 1:1 binding model, The U-values are representative of the confidence and validity of the results.[7]

S4.3.2. Control SPR sensograms of CB[7] tethered DNA

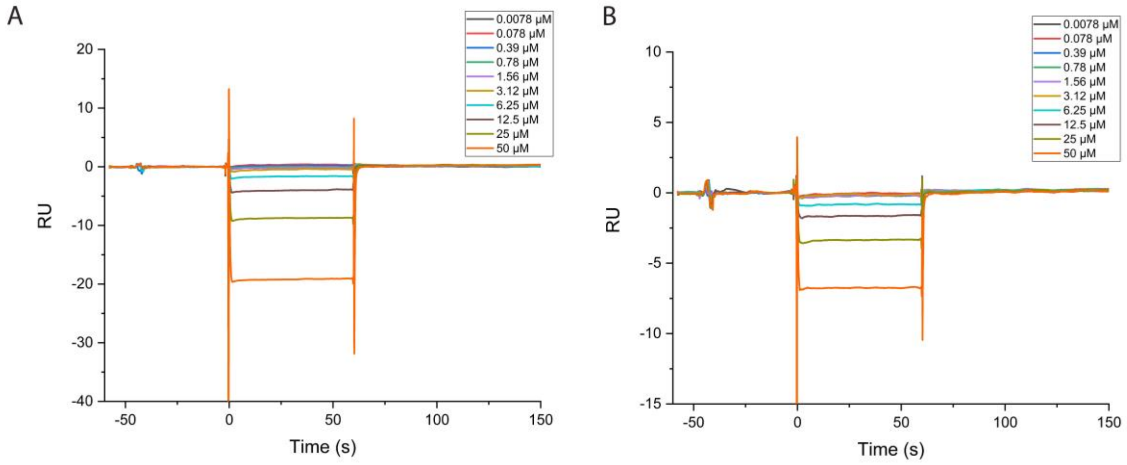

Supplementary Figure 33 ii(A) SPR of single-stranded CB[7] containing 12 mer **S** binding to hCA-II and (B) SPR of ds-CB[7] DNA binding to hCA-II. The concentration of DNA was varied from 0.0078 to 50 µM. The running buffer used in the SPR binding assays was PBS-P+ (containing 0.5% surfactant P20, pH 7.4). These controls show only negative SPR signals (after standard reference surface subtraction) indicating that the CB[7]-DNA has a higher affinity for the unmodified (no protein) surface over the hCA-II protein immobilized surface. Such signals are established in the literature.[8]

S4.3.3. Control experiments performed on hCA-II enzyme activity

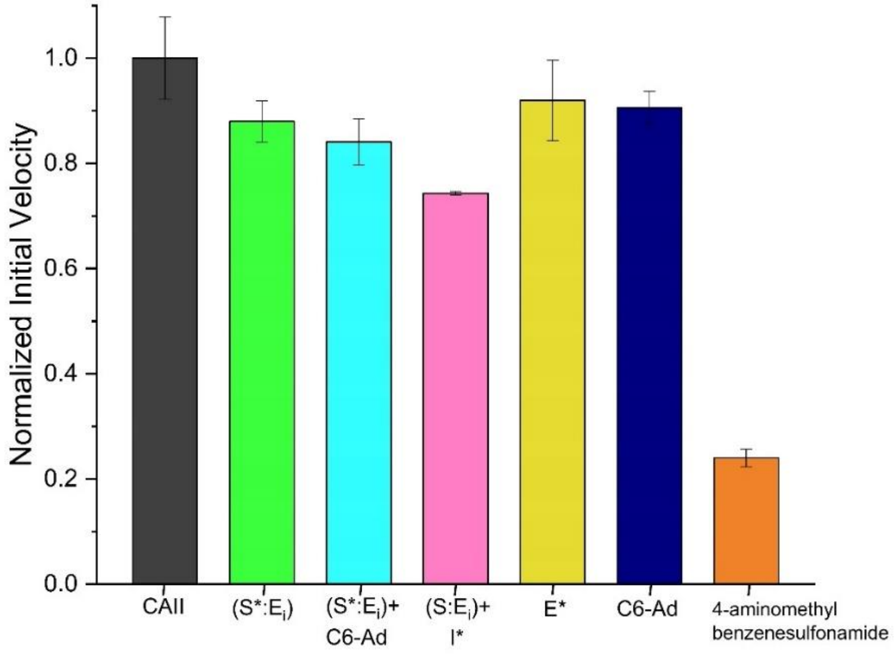

Supplementary Figure 34. Normalized hCA-II activity for control experiments performed at 5 µM concentration of inhibitors (with 25 µM of relevant input strands). The activity of hCA-II under various conditions tested was normalized to the blank (hCA-II without any inhibitors/ DNA strands, black). The buffer used in the inhibition/ displacement studies was: 20 mM Tris, 10 mM NaCl, 5 mM $MgCl_2$, pH = 7.5. Results are the mean ± SD of 3 independent experiments.

When h-CAII was incubated with the duplex **S*:R** without the CB7 host modification, 88% protein activity was obtained (green) whereas the **S:R** duplex containing CB7 had shown 83% activity (main text Figure 5D), indicating that the presence of CB7 had no significant effect on the hCA-II inhibition. When hCA-II was incubated with the modified DNA strand that lacks the benzenesulfonamide inhibitor headgroup (**E***), 92% activity was observed (yellow), indicating that the small molecule inhibitor is critical for hCA-II inhibition. Since the displacement assays were conducted with 5 times excess (25 µM) of **C6-Ad** fuel strands (compared to **S:Ei**), another important control study was performed by incubating hCA-II with 25 µM **C6-Ad**, and the activity was found to be 92% (dark blue), which indicates no significant inhibition due to excess of **C6-Ad** present in the medium.

Two additional control displacement studies were done in the presence of hCA-II, for the systems that lack the host-guest interactions. For the **S*:Ei** + **C6-Ad** (light-blue) that lacks CB7, and **S:Ei** + **I*** (pink) that lacks the guest modification, hCA-II activity was found to be 84% and 74% respectively. A positive control was

also conducted by using the small molecule inhibitor, 4-(aminomethyl) benzenesulfonamide that resulted in 24% hCA-II activity (the same level of activity observed for **Ei** (n=1); see main text Figure 5D-red bar). Taken together, these controls further confirm that the effect of hCA-II inhibition is due to the single stranded **Ei** binding to hCA-II via the sulfonamide headgroup and that the host-guest interactions are needed to facilitate the strand displacement.

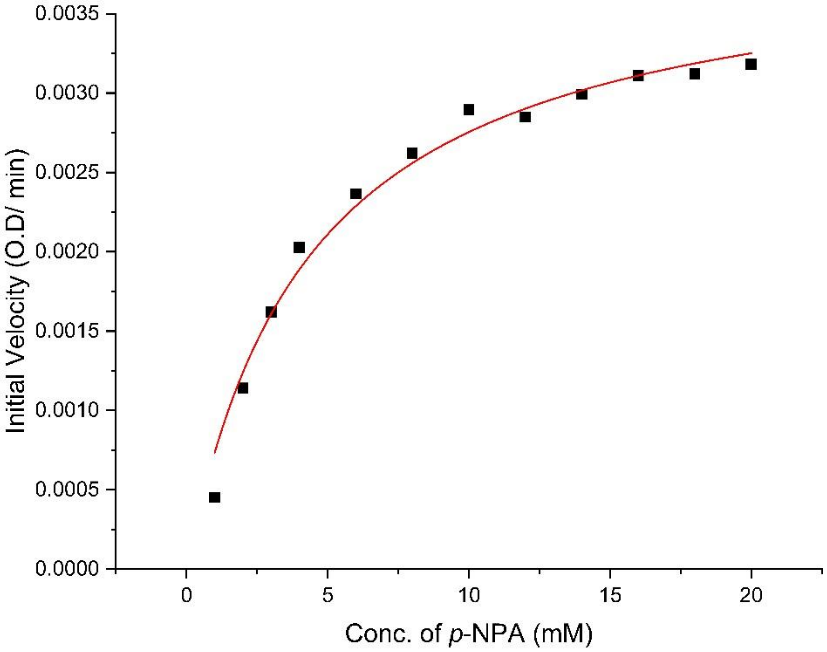

Supplementary Figure 35. Michaelis-Menten curve for p-NPA hydrolysis catalyzed by hCA-II at different concentrations of substrate. The buffer used in this study was: 20 mM Tris, 10 mM NaCl, 5 mM $MgCl_2$, pH = 7.5. The data was fit to the Michaelis Menten Equation (2).

$$V_0 = \frac{V_{max}[S]}{K_m + [S]} \qquad \textbf{(Eq. 2)}$$

Here, [S] is the total concentration of the substrate p-NPA, which is 1.0 mM. The Michaelis−Menten constant K_m (the substrate concentration that gives a reaction rate equal to one-half the maximum rate) was first obtained for p-NPA hydrolysis catalyzed by hCA-II in the absence of inhibitors by fitting the data (Supplementary Figure 35) to the Michaelis−Menten equation (2) where V_0 is the initial velocity and V_{max} is the maximum velocity of the reaction.

S4.4. Supplementary figures relevant to miR-182 detection

S4.4.1. Determination of Anchor duplex loading on MB

In order to determine the concentration of anchor duplex **A:C6-Ad** that was loaded onto the magnetic beads, we first prepared a duplex analog that was fluorescent. Specifically, a 5'-biotin and 3'-fluorescein modified anchor DNA strand **(A')** was first hybridized with **C6-Ad** in buffer (20 mM Tris, 10 mM NaCl, 5 mM $MgCl_2$, pH = 7.5, resulting in duplex **A':C6-Ad.** A dilution series (0.1- 0.6 nmol) of this duplex was then prepared and a calibration curve was constructed based on the fluorescence emission of the 3'-fluorescein unit on **A':C6-Ad** (*vide infra*).

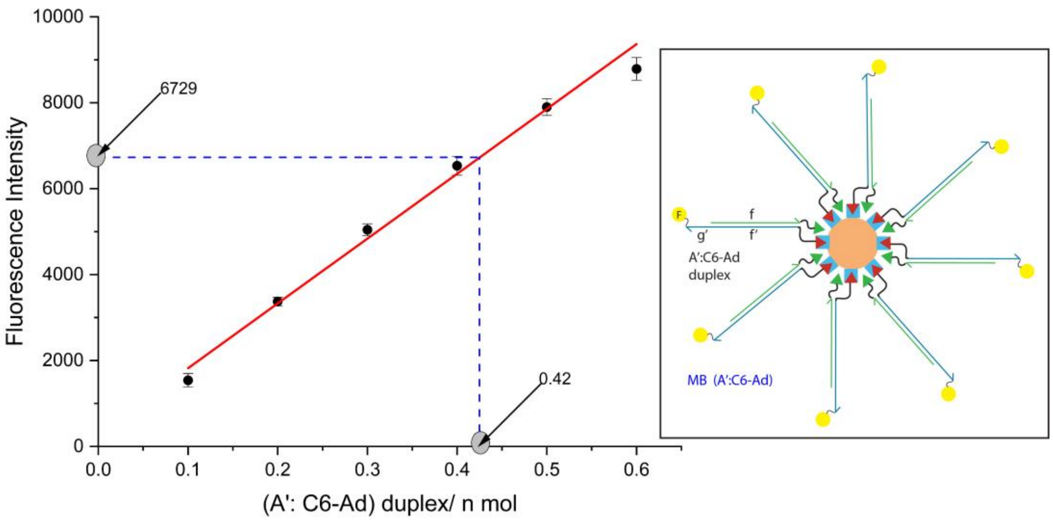

Supplementary Figure 36. Calibration curve to determine the loading of duplex (A':C6-Ad) bound on to magnetic beads (MB). Fluorescence intensity of **A':C6-Ad** (0.1-0.6 n mol) was measured at 522 nm emission wavelength**.** The buffer used in this study was: 20 mM Tris, 10 mM NaCl, 5 mM $MgCl_2$, pH = 7.5. Results are the mean ± SD of 3 independent experiments. An R^2 of 0.99 was obtained for the linear fit. **Inset:** Schematic of the magnetic beads decorated with fluorescent **A':C6-Ad** duplex.

The duplex **A':C6-Ad** was prepared by hybridizing 100 µL of 20 µM **A'** with 100 µL of 24 µM **C6-Ad**. This biotin-containing **A':C6-Ad** duplex (200 µL in assay buffer/ 2.0 nmol) was added to an eppendorf containing **MB** followed by incubation and gentle agitation using a mechanical shaker for 3 hours at RT. After removal of duplex-bound **MB**, the concentration of the remaining **A':C6-Ad** duplex in the supernatant solution was determined via fluorescence. The measured value was 6729 (mean of 3 independent experiments) which corresponds to 0.42 nmol of free non-bound duplex **A':C6-Ad**. Hence the amount of **A':C6-Ad** bound to **MB** was estimated to be 1.58 nmol (2.0 minus 0.42 nmol). From this experiment, it was assumed that when the non-fluorescent duplex, **A:C6-Ad,** is loaded onto the **MB (**used for the miR detection study, see main text Figure 6 and method section for details)**,** the loading was also 1.58 nmol.

S4.4.2. Determination LOD for miR-182 detection

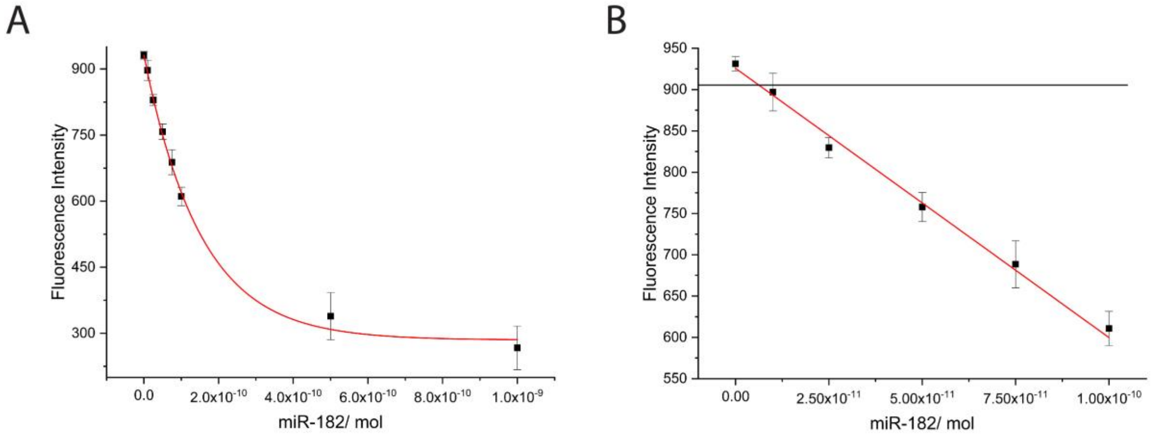

Supplementary Figure 37 (A). calibration curve was prepared by introducing varying amounts of miR-182 (0-1.0 nmol) into the magnetic bead displacement assay (see Figure 6A, main text). A LOD was determined using 3 SD below the averaged blank signal. (B) The linear portion of the graph was used to calculate the LOD value (using the linear fit Y= mX + C). The LOD was found to be 6.3 pmol. Buffer used in these studies: 20 mM Tris, 10 mM NaCl, 5 mM $MgCl_2$, pH = 7.5. Results are the mean ± SD of 3 independent experiments.

S5. Supplementary Tables

Note: **S*** and **I*** are the core, unmodified, DNA sequences lacking modifications for host and guest attachments (as well as linkers), respectively. **S'** and **I'** are core DNA sequences that have an internal 12 carbon spacer (X = spacer C12 CE phosphoramidite) within the sequence. **S"** and **I"** are core DNA sequences without any modifications.

S5.1. DNA sequences used in the development of HG-TMSD

DNA Strand	***Core DNA sequence 5'-3'***
S*	GGC TAC ACT AGA AGG ACA GTA TTT G
I*	CAA ATA CTG TCC TTC TAG TGT AGC C
S"	ACT GCA GGC TAC ACT AGA AGG ACA GTA TTT G
I"	CAA ATA CTG TCC TTC TAG TGT AGC CTG CAG T
S'	ACT GCA **X** GGC TAC ACT AGA AGG ACA GTA TTT G
I'	CAA ATA CTG TCC TTC TAG TGT AGC C **X** TGC AGT
S	GGC TAC ACT AGA AGG ACA GTA TTT G
R	CAA ATA CTG TCC TTC TAG TGT AGC C
C6-Ad or C6-Phe	CAA ATA CTG TCC TTC TAG TGT AGC C
EG3 C6-Ad or EG3 C6-Phe	CAA ATA CTG TCC TTC TAG TGT AGC C
EG6 C6-Ad or EG6 C6-Phe	CAA ATA CTG TCC TTC TAG TGT AGC C
EG9 C6-Ad or EG9 C6-Phe	CAA ATA CTG TCC TTC TAG TGT AGC C
EG12 C6-Ad or EG12 C6-Phe	CAA ATA CTG TCC TTC TAG TGT AGC C
CS	TGC AGT

Supplementary Table 1. DNA sequences used to develop and modulate the HG-TMSD mechanism.

S5.2. DNA sequences used in the DNA machines that utilize HG-TMSD to control hCA-II activity

DNA Strand	***Core DNA sequence 5’-3’***
S*	GGT GGA CGG GCC
I*	GGC CCG TCC ACC
E*	GGC CCG TCC ACC
S	GGT GGA CGG GCC
C6-Ad or C6-Phe	GGC CCG TCC ACC
Ei (n=1) or Ei (n=2)	GGC CCG **T**CC ACC<br>(**T** is decorated with the inhibitor molecule)

Supplementary Table 2. DNA sequences used for regulation of hCA-II activity via HG-TMSD.

S5.3. DNA sequences used in the sequential BP-TMSD and HG-TMSD reactions to detect miR-182

DNA Strand	***Core DNA sequence 5’-3’***
S*	AGT GTG AGT TCT ACC ATT
I*	AAT GGT AGA ACT CAC ACT
miR-182	UUU GGC AAU GGU AGA ACU CAC ACU
miR-183	UAU GGC ACU GGU AGA AUU CAC U
miR-381	UAU ACA AGG GCA AGC UCU CUG U
S	AGT GTG AGT TCT ACC ATT
R	AAT GGT AGA ACT CAC ACT
C6-Ad	AAT GGT AGA ACT CAC ACT
A	AGT GTG AGT TCT ACC ATT GCC AAA
A’	AGT GTG AGT TCT ACC ATT GCC AAA

Supplementary Table 3. DNA sequences used to develop BP-TMSD and HG-TMSD based layered reaction that enables detection of miRNA-182, via fluorescence-quenching.

S5.4. Input DNA sequences used in HG-TMSD mutation studies

DNA Strand	***Core DNA sequence 5'-3'***
M3 C6-Ad	CAA ATA CTG TCC TTC TAG TGT ATC C
M14 C6-Ad	CAA ATA CTG TCA TTC TAG TGT AGC C
M25 C6-Ad	AAA ATA CTG TCC TTC TAG TGT AGC C

Supplementary Table 4. Input DNA sequences used in the mutation studies with mutant location marked in red.

S5.5. DNA sequences used in thermal denaturation experiments

DNA Strand	***Core DNA sequence 5'-3'*** 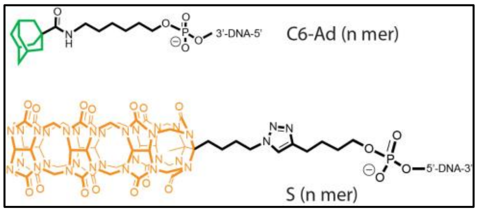
S (25 mer)	GGC TAC ACT AGA AGG ACA GTA TTT G
C6-Ad (25 mer)	CAA ATA CTG TCC TTC TAG TGT AGC C
S (8 mer)	GGC TAC AC
C6-Ad (8 mer)	GTG TAG CC
S (7 mer)	GGC TAC A
C6-Ad (7 mer)	TGT AGC C
S (5 mer)	GGC TA
C6-Ad (5 mer)	TAG CC
S (4 mer)	GGC T
C6-Ad (4 mer)	AGC C

Supplementary Table 5. DNA sequences used in the thermal denaturation studies.

S5.6. RP-HPLC eluent gradient

Time/ min	***Flow (mL/min)***	***%A***	***%B***
0.00	0.75	100	0
0.01	1.00	100	0
2.50	1.00	100	0
12.50	1.00	92	8
25.00	1.00	80	20
34.00	1.00	50	50
36.00	1.00	0	100
38.00	1.00	0	100
40.00	1.00	100	0
42.00	1.00	100	0

Supplementary Table 6. HPLC eluent gradient used for DNA conjugate purification. Solvent A is 0.1 M TEAA (aq) in 5% acetonitrile and solvent B is 100% acetonitrile.

S6. Thermal Denaturation Studies of Host-Guest Stabilized DNA Duplexes

S6.1. Synthesis and characterization of DNA-small molecule conjugates

DNA sequences used in this set of experiments are mentioned in the Supplementary Table 5.

Synthesis of all CB[7] conjugated DNA sequences (**S**; 25mer/ 8mer/ 7mer/ 5mer/ 4mer) followed the synthetic scheme shown in Supplementary Figure 1. Synthesis of all adamantane conjugated DNA sequences followed the synthetic scheme shown in the Supplementary Figure 2. All DNA conjugates were RP-HPLC purified and characterized with MALDI-TOF.

MALDI-TOF observed MW of **S** 8mer was 3827.7 Da, while the calculated MW was 3829.9 Da for $[M+H]^+$. MALDI-TOF observed MW of **S** 7mer was 3538.0 Da, while the calculated MW was 3540.7 Da for $[M+H]^+$. MALDI-TOF observed MW of **S** 5mer was 2935.7 Da, while the calculated MW was 2938.3 Da for $[M+H]^+$. MALDI-TOF observed MW of **S** 4mer was 2622.6 Da, while the calculated MW was 2625.1 Da for $[M+H]^+$.

MALDI-TOF observed MW of 8mer **C6-Ad** was 2764.3 Da, while the calculated MW was 2768.0 Da for $[M+H]^+$. MALDI-TOF observed MW of 7mer **C6-Ad** was 2435.5 Da, while the calculated MW was 2438.8 Da for $[M+H]^+$. MALDI-TOF observed MW of 5mer **C6-Ad** was 1803.8 Da, while the calculated MW was 1805.4 Da for $[M+H]^+$. MALDI-TOF observed MW of 4mer **C6-Ad** was 1499.1 Da, while the calculated MW was 1501.2 Da for $[M+H]^+$.

S6.2. General experimental of thermal denaturation studies

UV-Vis spectroscopy followed melting curves of the duplexes were collected in buffer containing 20 mM Tris, 10 mM NaCl, and 5 mM $MgCl_2$ at pH = 7.5. Spectra were collected in a closed capped quartz cuvette, which had a pathlength 1 cm. Absorbance was monitored at 260 nm while ramping the sample from 20°C to 90°C and back to 20°C at a rate of 2 degrees per minute. All duplexes were denatured at a concentration of 1 uM and denaturation experiments for each duplex were performed in triplicate. The melting temperature was determined using a sigmoidal fit. Predicted denaturation temperatures for unmodified (i.e., no host or guest) duplexes were calculated using the OligoAnalyzer tool (Integrated DNA Technologies) software.

S6.3. Results and discussion of thermal denaturation studies of host-guest stabilized DNA duplexes

Thermal denaturation experiments were performed on the 25mer duplex **S:I** (where **I** is **C6-Ad**) to determine the stabilizing effects of the host-guest interactions.

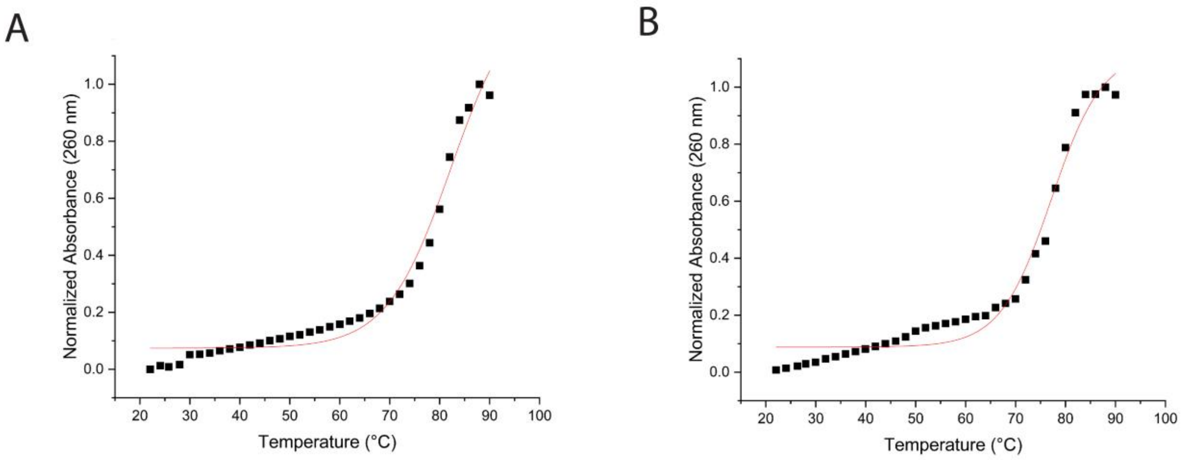

Supplementary Figure 38. (A) Representative thermal denaturation profiles for **S:I** where **I** is **C6-Ad** (25 mer) at a concentration of 1 µM in buffer containing 20 mM Tris, 10 mM NaCl, and 5 mM $MgCl_2$ at pH 7.5. (B) Representative renaturation profile for **S + C6-Ad** at a concentration of 1 µM in buffer containing 20 mM Tris, 10 mM NaCl, and 5 mM $MgCl_2$ at pH 7.5. Experiments were performed in triplicate.

As can be seen from Supplementary Figure 38(A), the CB[7]-adamantane interaction significantly stabilizes the 25-mer duplex. The melting temperature (T_m) was found to be 80.9°C. This is significantly enhanced (by 15.2°C) relative to the predicted transition for the corresponding unmodified 25-mer (see Supplementary Table 7). We also performed renaturation studies (Supplementary Figure 38(B) to make sure that the duplex is fully dissociated (and an upper baseline is achieved) at 90 °C. The T_m of 80.9°C is remarkably stable and corresponds, for e.g., to a conventional unmodified duplex consisting of over 69 base pairs with GC content of 52.2%.

Thermal denaturation experiments were performed on host-guest stabilized shorter duplexes.

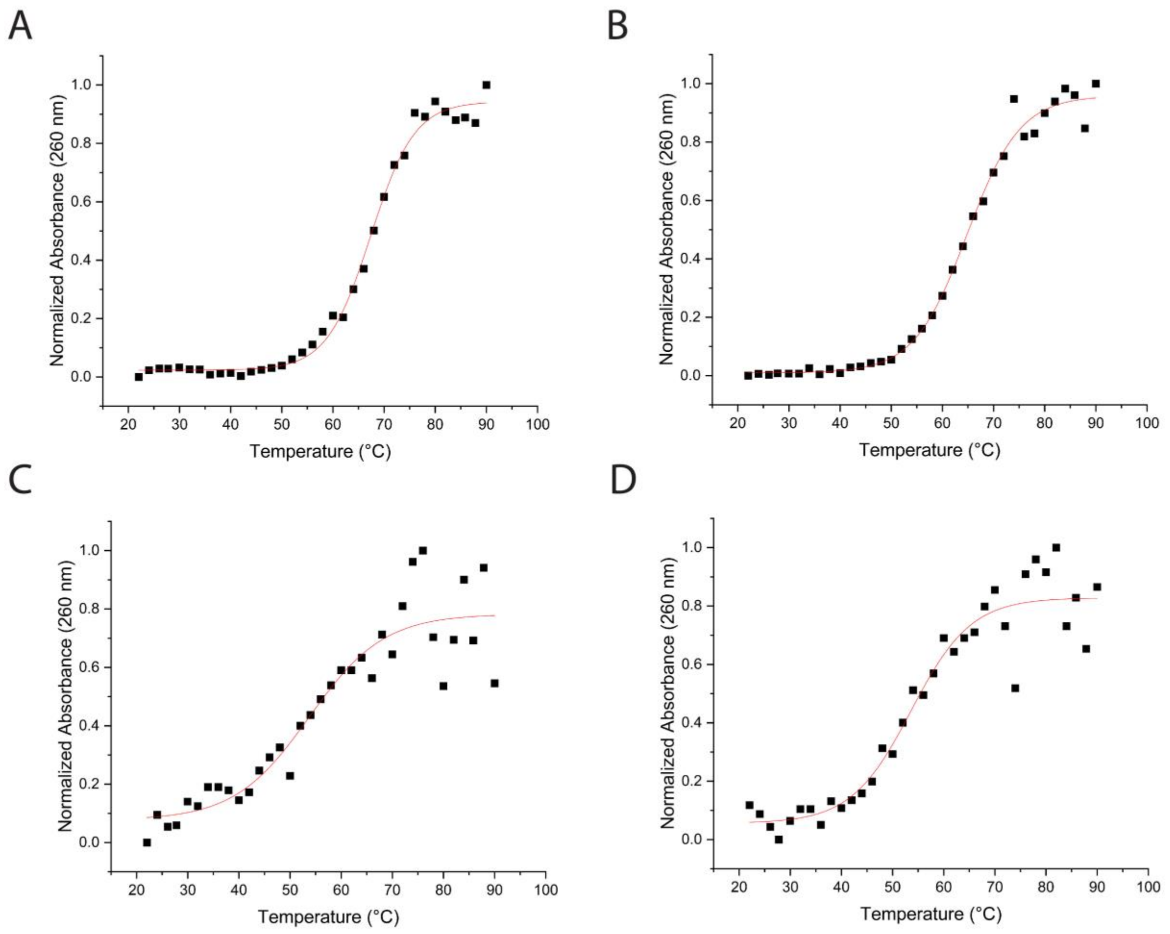

Supplementary Figure 39. Representative thermal denaturation profiles for **S:C6-Ad** duplex composed of (A) 8 mer, (B) 7 mer, (C) 5 mer, and (D) 4 mer. Concentration of duplexes were 1 µM in buffer containing 20 mM Tris, 10 mM NaCl, and 5 mM $MgCl_2$ at pH 7.5. Experiments were performed in triplicate.

In order to determine whether host-guest interactions can be used to stabilize otherwise unstable duplexes at ambient temperature, thermal denaturation experiments were also carried out on CB[7]-adamantane containing duplexes consisting of short sequences (Supplementary Table 7). Interestingly, the stability of each of these duplexes is enhanced significantly in the presence of the host-guest interaction, with shorter duplexes showing increasing ΔT_m. Further, for very short duplexes, consisting of 5 or 4 base pairs (see Supplementary Figure 39 C & D) that are conventionally completely unstable at room temperature (are melted at 0 °C), we still observe an appreciable melting profile upon addition of the host-guest interaction. The T_ms for these duplexes coalesce at ~ 53°C. This finding suggests that these duplexes are

predominantly stabilized by the host-guest interaction. Note: the data for the 4 and 5 base pair containing duplexes display scattering, especially at temperatures above ~ 70°C. This is likely due to noise arising from the fact that there is low absolute change in absorption going from the stacked to un-stacked state for such very short duplexes.

Supplementary Table 7.

Melting Temperature (T_m) of DNA Duplexes (1 µM) (°C)					
	4-mer	5-mer	7-mer	8-mer	25-mer
No Host-Guest Interaction (Predicted)*	0	0	25.7	34.1	65.7
With Host-Guest Interaction (Average of Triplicates)	52.5 ± 0.9	52.7 ± 0.9	64.8 ± 0.1	67.2 ± 0.3	80.9 ± 1.3
ΔT_m	52.5 ± 0.9	52.7 ± 0.9	39.1 ± 0.1	33.1 ± 0.3	15.2 ± 1.3

*Predicted denaturation temperatures for unmodified (i.e., no host or guest) duplexes were calculated using the OligoAnalyzer tool (Integrated DNA Technologies) software.

S7. References

1. Zhou, M.; Haldar, S.; Franses, J.; Kim, J.-M.; Thompson, D. H., Synthesis and Self-assembly Properties of Acylated Cyclodextrins and Nitrilotriacetic Acid (NTA)-modified Inclusion Ligands for Interfacial Protein Crystallization. Supramolecular Chemistry 2006, 17 (1-2), 101-111.
2. Vinciguerra, B.; Cao, L.; Cannon, J. R.; Zavalij, P. Y.; Fenselau, C.; Isaacs, L., Synthesis and self-assembly processes of monofunctionalized cucurbit[7]uril. J Am Chem Soc 2012, 134 (31), 13133-40.
3. Zuker, M., Mfold web server for nucleic acid folding and hybridization prediction. Nucleic Acids Res 2003, 31 (13), 3406-15.
4. Brandt, R. B.; Laux, J. E.; Yates, S. W., Calculation of inhibitor Ki and inhibitor type from the concentration of inhibitor for 50% inhibition for Michaelis-Menten enzymes. Biochem Med Metab Biol 1987, 37 (3), 344-9.
5. Khodakov, D. A.; Khodakova, A. S.; Huang, D. M.; Linacre, A.; Ellis, A. V., Protected DNA strand displacement for enhanced single nucleotide discrimination in double-stranded DNA. Sci Rep 2015, 5, 8721.
6. Genot, A. J.; Zhang, D. Y.; Bath, J.; Turberfield, A. J., Remote toehold: a mechanism for flexible control of DNA hybridization kinetics. J Am Chem Soc 2011, 133 (7), 2177-82.
7. Sabapathy, T.; Deplazes, E.; Mancera, R. L., Revisiting the Interaction of Melittin with Phospholipid Bilayers: The Effects of Concentration and Ionic Strength. Int J Mol Sci 2020, 21,746.
8. Atale, S. S.; Dyawanapelly, S.; Jagtap, D. D.; Jain, R.; Dandekar, P., Understanding the nano-bio interactions using real-time surface plasmon resonance tool. Int J Biol Macromol 2019, 123, 97-107.
